# Bypassing stroke-damaged neural pathways via a neural interface induces targeted cortical adaptation

**DOI:** 10.1038/s41467-019-12647-y

**Published:** 2019-10-16

**Authors:** Kenji Kato, Masahiro Sawada, Yukio Nishimura

**Affiliations:** 1 Department of Developmental Physiology, National Institute for Physiological Sciences, 38 Nishigonaka, Myodaiji, Okazaki, Aichi 444-8585 Japan; 2 Department of Physiological Sciences, School of Life Science, The Graduate University for Advanced Studies, SOKENDAI, Shonan Village, Hayama, Kanagawa 240-0193 Japan; 3 Japan Society for The Promotion of Science, Kojimachi Business Center Building, 5-3-1 Kojimachi, Chiyoda, Tokyo 102-0083 Japan; 4 Department of Neurosurgery, Graduate School of Kyoto University, 54 Shogoin-kawaharacho, Sakyo, Kyoto 606-8507 Japan; 5 Neural Prosthesis Project, Department of Dementia and Higher Brain Function, Tokyo Metropolitan Institute of Medical Science, 2-1-6, Kamikitazawa, Setagaya, Tokyo 158-8506 Japan; 6 Department of Neuroscience, Graduate School of Medicine and Faculty of Medicine, Kyoto University, Yoshida-Konoe, Sakyo, Kyoto 606-8501 Japan; 7 Precursory Research for Embryonic Science and Technology, Japan Science and Technology Agency, Sanban-tyo, Chiyoda, Tokyo 102-0076 Japan; 8 Present Address: Center of Assistive Robotics and Rehabilitation for Longevity and Good Health, National Center for Geriatrics and Gerontology, 7-430, Morioka, Obu, Aichi 474-8511 Japan

**Keywords:** Neuroscience, Medical research, Engineering

## Abstract

Regaining the function of an impaired limb is highly desirable in paralyzed individuals. One possible avenue to achieve this goal is to bridge the interrupted pathway between preserved neural structures and muscles using a brain–computer interface. Here, we demonstrate that monkeys with subcortical stroke were able to learn to use an artificial cortico-muscular connection (ACMC), which transforms cortical activity into electrical stimulation to the hand muscles, to regain volitional control of a paralysed hand. The ACMC induced an adaptive change of cortical activities throughout an extensive cortical area. In a targeted manner, modulating high-gamma activity became localized around an arbitrarily-selected cortical site controlling stimulation to the muscles. This adaptive change could be reset and localized rapidly to a new cortical site. Thus, the ACMC imparts new function for muscle control to connected cortical sites and triggers cortical adaptation to regain impaired motor function after stroke.

## Abstract (web summary)

Monkeys were trained to use an artificial cortico-muscular connection (ACMC) to regain control over a paralyzed hand following subcortical stroke. Control over the paralyzed hand was accompanied by the appearance of localized high-gamma modulation in the cortex, which could be rapidly reset and relocalized to a different cortical site to reactivate motor control.

## Introduction

Paralysis following stroke is a leading cause of long-term motor disability. Up to 30% of stroke survivors must depend on assistance to manage their daily living activities. Therefore, restoration of lost motor function is a high priority issue^1,2^. Brain machine interfaces (BMIs) can transform cortical activity into control signals for an external device, such as a robotic arm or computer cursor, and may provide a solution for restoring lost function^3–8^.

Patients who suffer a stroke or spinal cord injury, where a limb is paralysed but intact, have an intense desire to regain function of the impaired limb. Bypassing the damaged pathway using brain-controlled functional electrical stimulation (FES) to regain volitional control of the paralysed limb is promising for restoring lost motor function^9–17^. Brain-controlled FES works as an “artificial” neural pathway by creating a causal relationship between brain activity and an evoked limb movement. However, subjects may be required to learn a novel causal input-output relationship to control the paralysed limb.

Disruption of descending pathways results in a lost connection between the brain and target muscles. Functional recovery in such a situation is characterised by substantial reorganisation in the structure and function of the damaged brain^18–22^. Thus, our nervous system shows remarkable flexibility to adapt to novel neuromotor mappings. How the brain incorporates a novel “artificial” neural pathway into volitional limb control within the surviving cortical areas remains largely unclear.

In the present study, we generated a model of chronic hemiparalysis in the extremities caused by subcortical stroke in monkeys. We then employed an artificial cortico-muscular connection (ACMC) to connect the preserved cortical areas to muscles beyond the damaged site. Specific neural oscillations in the cortical area were detected contingent to the input and converted into electrical stimulation delivered to the muscles in real time. We demonstrated that, despite damage to subcortical areas, a flexible change in the neural oscillations controlling the ACMC was observed in a targeted manner throughout an extensive sensorimotor area. Thus, monkeys that experienced a subcortical stroke could rapidly learn to regain lost volitional control of a paralysed hand.

## Results

### A primate subcortical stroke model

Three macaque monkeys were used in this study: two monkeys (Monkeys M and TA) were trained to perform visually-guided center-out position and/or wrist-torque-tracking tasks, and one monkey (Monkey TE) was not trained on these tasks. Following implantation of a subdural electrocorticogram (ECoG) electrode grid over the frontal eye field (FEF), premotor (PM), primary motor (M1), and primary somatosensory (S1) areas, or a microelectrode array over M1, focal ischaemia was induced at the level of the corona radiate by embolization of the lenticulostriate and/or anterior coronary arteries. The lesion extended into extensive subcortical areas, including the cerebral peduncle, internal capsule, caudate, and putamen; therefore, most descending and ascending pathways were severely damaged. However, the cerebral cortex appeared to be largely intact (Supplementary Fig. 1). Immediately after stroke induction, two monkeys (Monkeys TE and TA) displayed nearly complete motor hemiparalysis, with a somatosensory deficit of the face and upper and lower extremities on the side contralateral to the lesion. One monkey (Monkey M) showed nearly complete motor and somatosensory hemiparalysis in only the upper extremity on the side contralateral to the lesion. Monkeys showed a gradual improvement of voluntary hand control without the ACMC. Even in this period, the monkeys displayed severe paresis of wrist and finger movements, which were not functional in daily life. All experiments were performed before these spontaneous recovery appeared. Experiments in Monkeys TA, TE and M were performed until post-day 38, 105 and 24 respectively.

### Volitional control of the paralysed hand via an ACMC

**Fig. 1:**
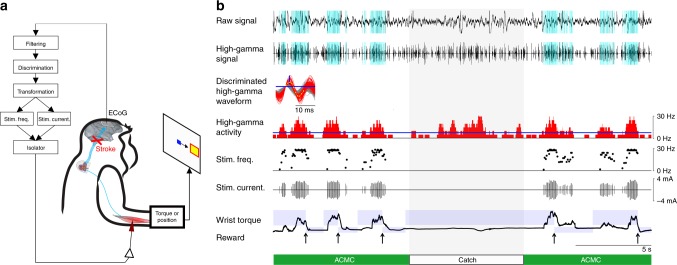
Volitional control of a paralysed hand via an artificial cortico-muscular connection. **a** Each monkey controlled the position of a cursor (blue square) using wrist torque or hand position to acquired targets (red square) displayed on the screen. An artificial cortico-muscular connection (ACMC) was achieved by converting cortical oscillatory activity in real time to electrical stimulation of paralysed muscles. **b** A example of five successful trials from a torque-tracking task when the ACMC was on (green) and one catch trial when the ACMC was switched off (grey shading) in Monkey TA. Extensor and centre wrist torque targets (blue shading) were presented on the screen. The input microelectrode was located in the hand area of the primary motor cortex (M1), and electrical stimuli were applied to the extensor carpi radialis (ECR) and extensor digitorum communis (EDC) muscle pair. A raw signal (1st row) was recorded from the electrode in M1 and filtered in the high-gamma frequency band (2nd row). The blue vertical lines in the 1st and 2nd rows indicate the timing of electrical stimulation. An arbitrary one-cycle high-gamma waveform was selected (3rd row) from the high-gamma oscillations as the input signal for controlling the ACMC. The red traces in the 3rd row indicate the high-gamma waveforms detected by the template-matching method, with the thresholds represented as blue lines in the 3rd row. The yellow lines with 10 yellow dots in the 3rd row represent the template for high-gamma waveform detection. The 4th row indicates the frequencies of the detected high-gamma waveforms. Electrical stimulation was delivered to the paralysed ECR and EDC muscles with a current (5th trace) and frequency (6th trace) that were proportional to the moving averaged frequencies (250-ms time window) of the detected high-gamma waveforms above a stimulation threshold (blue line in the 4th trace). The blue rectangles in the wrist torque trace in the 7th row represent the extensor and centre wrist torque targets. The arrows at the bottom indicate successful trial completion and reward times. Similar results were obtained from independent experiments (*N* = 3 monkeys, *n* = 108 sessions)

To regain volitional control of the paralysed hand, an ACMC was used. The ACMC, accomplished using a brain-computer interface, was designed to detect a high-gamma oscillatory activity pattern (80–120 Hz) from a single electrode and convert it in real time to activity-contingent electrical stimulation to the muscles (Fig. 1a). An arbitrary one-cycle waveform was selected from high-gamma oscillations using a template-matching algorithm to identify a particular shape and amplitude of waveforms (Supplementary Fig. 2). High-gamma waveforms with relatively high amplitude that could be reliably distinguished from stimulus artefacts were used for the ACMC input signal (Supplementary Fig. 2 and see also 3^rd^ row in Fig. 1b). The stimulation current and frequency to the muscles were proportional to the frequency of the high-gamma episodes; thus, the monkeys could voluntarily control the current and frequency of the electrical stimulation by altering a single high-gamma activity source (Fig. 1a; see the Methods). Figure 1b shows an example of typical torque-tracking task performance in a primate sub-cortical stroke model connected to the ACMC, which bridged an M1 site and the wrist extensor muscles (extensor carpi radialis [ECR] and extensor digitorum communis [EDC]). The monkey was able to volitionally modulate the high-gamma activity to control stimulation to the muscles via the ACMC (green bar in Fig. 1b); thus, the monkey could acquire the targets repeatedly. To confirm the efficacy of the ACMC, it was turned off during a brief “catch trial” period (white bar in Fig. 1b). During the catch trial, the monkey continued to increase the high-gamma activity; however, it was unable to acquire the targets due to severe paralysis, indicating that the ACMC was necessary for the volitional control of wrist torque. In almost half of the ACMC sessions, the monkeys also successfully performed a three-graded torque- or position-tracking task (Supplementary Fig. 3). Moreover, although one monkey (Monkey TE) had not previously been trained in the visual-guided position and torque-tracking tasks, it acquired the target rapidly during ACMC application, similar to the other well-trained monkeys (see Supplementary Fig. 4a).

**Fig. 2:**
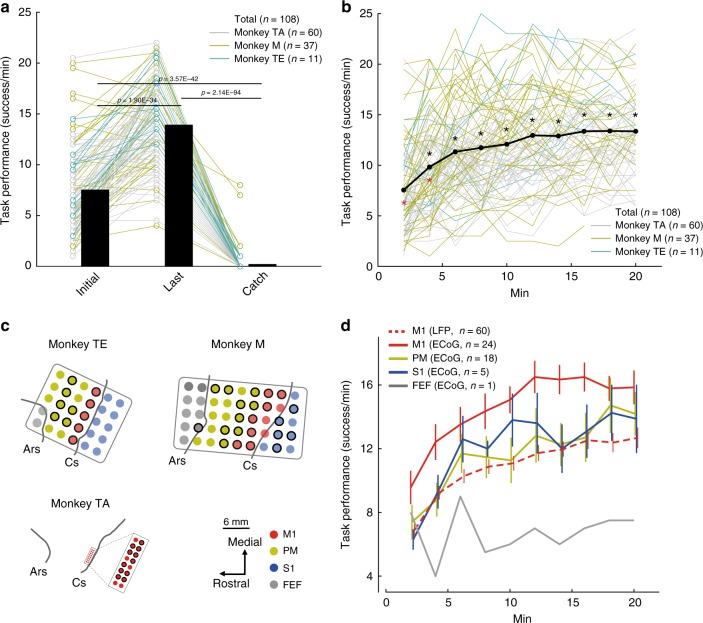
Task performance improves with practice. **a** Task performance during the initial and last 2 min of a session and during the catch trials (*N* = 3 monkeys, *n* = 108 ACMC sessions, consisting of 72 torque-matching and 36 position-matching task sessions; Monkey TA, *n* = 60 sessions; Monkey M, *n* = 37 sessions; Monkey TE, *n* = 11 sessions). Bars indicate mean values and circles indicate individual data points. **b** Time course of averaged task performance across 108 sessions in the three monkeys; Black dots show mean values; thin lines represent individual sessions. Black and red asterisks represent significant differences (*P* < 0.05 by one-way ANOVA with Bonferroni’s correction for post hoc multiple comparisons) compared to the initial and last 2 min of the session, respectively. **c** Electrode locations. In Monkeys TE and M, the electrocorticography (ECoG) electrode array was placed on the surface of the frontal eye field (FEF; grey), premotor cortex (PM; yellow), primary motor cortex (M1; red), and primary somatosensory cortex (S1; blue). In Monkey TA, 16 microelectrodes were implanted within the hand area of M1 (red dots). The electrodes that were used as the input signal for the ACMC are encircled in black. Ars: arcuate sulcus, CS: central sulcus. **d** Time course of task performance using the ACMC with input electrodes in the FEF (grey line, *n* = 1 session in Monkey M), PM (yellow line, *n* = 18 sessions in Monkey TE and M), M1 with ECoG array (red solid line, *n* = 24 sessions in Monkey TE and M), M1 with microelectrode array (dashed red line, *n* = 60 sessions in Monkey TA), and S1 (blue line, *n* = 5 sessions in Monkey M). Task performance of M1 electrodes with ECoG array was significantly higher than that with the other areas, and task performance with FEF electrode was significantly lower than that with the other areas (*P* < 0.05 by two-way ANOVA with Bonferroni’s correction for Post hoc multiple comparisons). Detailed statistical results for (**b**), (**d**) are shown in the source data. Data in (**d**) are shown as means and standard errors

In total, the three monkeys performed the tasks in 108 sessions (session duration: 6–74 min; trial number range within each session: 55–965), using 92 different pairs of cortical sites and muscles. The average task performance in the ACMC trials across monkeys was substantially higher than in the catch trials. 95/108 sessions showed task improvement. The average task performance in the last phase across monkeys was higher than that in the initial phase (Fig. 2a; One-way ANOVA; *F*_(2, 321)_ = 448.38, *P* = 1.15 × 10^−93^, post hoc multiple comparisons with Bonferroni’s correction; *P*_Initial vs Catch_ = 3.57 × 10^−42^; *P*_*Last vs Catch*_ = 2.14 × 10^−94^, *P*_Initial vs Last_ = 1.90 × 10^−34^; see also Supplementary Fig. 4a for the data of each monkey), suggesting that the ACMC was essential for the voluntary control of the paralysed limb and task improvement. Task performance in torque-tracking tasks was higher than that in position-tracking task (Supplementary Fig. 5a; two-way ANOVA; [task] *F*_(1, 318)_ = 7.53, *P* = 6.42 × 10^−3^, Supplementary Fig. 5b; two-way ANOVA; [task] *F*_(1, 906)_ = 23.73, *P* = 1.30 × 10^−6^). Task performance was higher in the two-graded tracking task than in the three-graded tracking task (Supplementary Fig. 5c; two-way ANOVA; [task] *F*_(1, 318)_ = 40.57, *P* = 6.64 × 10^−10^, Supplementary Fig. 5d; two-way ANOVA; [task] *F*_(1, 906)_ = 21.92, *P* = 3.12 × 10^−26^). In addition, task performance improved within 20 min from the start of the ACMC trial. A significant increase in task performance occurred within the initial 2 min and plateaued after 6 min (Fig. 2b, One-way ANOVA; *F*_(9,916)_ = 18.82, *P* = 5.11 × 10^−29^. Black and red asterisks indicate significant differences to performance successes in the initial 2 min and the last 20 min, respectively. *P* < 0.05 by one-way ANOVA with Bonferroni’s correction for post hoc comparisons. See also Supplementary Fig. 4b for results of each monkey). In each session, the input electrode for controlling the ACMC was selected randomly among widely distributed cortical areas (Fig. 2c). Monkeys were able to improve task performance irrespective of the location of the input electrode throughout the somatosensory-motor cortex, not only in M1, but also in S1 and PM. However, Monkey M failed to improve task performance when the input electrode was in the FEF (Supplementary Fig. 6, M1: one-way ANOVA; *F*_(2,249)_ = 100.49, *P* = 1.01 × 10^−32^. PM: one-way ANOVA; *F*_(2,51)_ = 14.44, *P* = 1.07 × 10^−5^. S1: one-way ANOVA; *F*_(2,12)_ = 15.44, *P* = 4.81 × 10^−4^. See also Fig. 2d). Before stroke, the somatotopic map was identified by electrical stimulation through the electrode within M1 in two monkeys (Monkeys TA and M). The electrodes in M1 were located in different somatotopic sites including wrist and non-wrist (face, digit, elbow, and shoulder) sites. Task improvement and peak performance were identical irrespective of the original somatotopy of M1 before stroke (Supplementary Fig. 7, two-way ANOVA; [Somatotopy] *F*_(1, 228)_ = 2.32, *P* = 0.13. not significant). Moreover, two monkeys (TE and M) controlled the ACMC using an oscillatory signal recorded with the ECoG array in the PM, M1, and S1 areas, while one monkey (TA) controlled the ACMC using an oscillatory signal recorded with a penetrating electrode array in M1. For electrodes in M1, task performance with ECoG electrodes was significantly higher than that with penetrating electrodes (Fig. 2d, Two-way ANOVA; [area] *F*_(4,876)_ = 30.44, *P* = 9.55 × 10^−24^, post hoc multiple comparisons with Bonferroni’s correction; *P*_M1 ECoG vs M1 LFP_ = 1.65 × 10^−22^).

### Volitional modulation of cortical activity from various regions

**Fig. 3:**
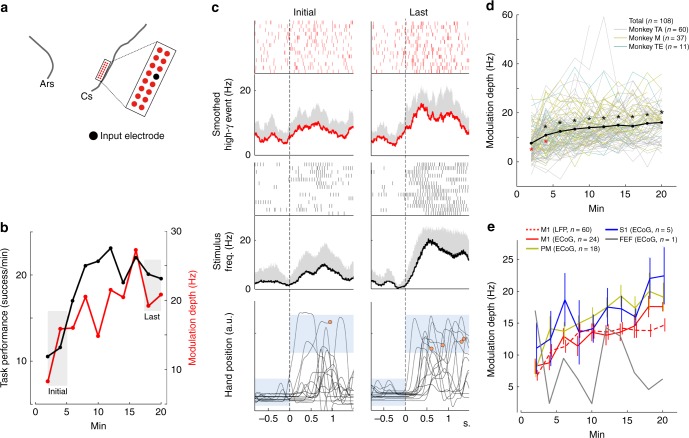
Changes in the pattern of high-gamma signal input during learning with an ACMC. **a**–**c** High-gamma activity of LFPs recorded from a penetrating electrode to grade muscle stimulation delivered to the ECR and EDC muscle pair in Monkey TA. **a** The black circle indicates the input electrode. **b** Representative time course of task performance (black line) and modulation depth (MD) of high-gamma activity at the input electrode site (red line). **c** Ensemble average of the raster plots of high-gamma activity, electrical stimulation, and hand position during the initial and last 14 trials. Plots are aligned to the time of target appearance, indicated by the vertical dotted lines. From the top, raster plot and averaged rate of occurrence of high-gamma activity, stimulus frequency, and hand position. The blue-shaded rectangles in the bottom trace represent the hand position targets. The orange dots represent the timing of successful trial completion and reward. **d** Average MDs measured at input electrodes (*N* = 3 monkeys, *n* = 108 sessions, consisting of 72 torque-matching and 36 position-matching task sessions; Monkey TA, *n* = 60 sessions; Monkey M, *n* = 37 sessions; Monkey TE, *n* = 11 sessions). Black dots indicate means; lines represent individual sessions. Black and red asterisks represent significant differences (*P* < 0.05 by one-way ANOVA with Bonferroni’s correction for post hoc multiple comparisons) compared to the initial and last 2 min, respectively. **e** Average MDs when input electrode was in the FEF (grey line, *n* = 1 session in Monkey M), PM (yellow line, *n* = 18 sessions in Monkey TE and M), M1 with ECoG array (red solid line, *n* = 24 sessions in Monkey TE and M), M1 with microelectrode array (dashed red line, *n* = 60 sessions in Monkey TA), and S1 (blue line, *n* = 5 sessions in Monkey M). MDs at the PM ECoG and S1 ECoG were significantly higher than MDs at the M1 LFP and FEF ECoG (*P* < 0.05 by two-way ANOVA with Bonferroni’s correction for post hoc multiple comparisons). Detailed statistical results for (**d**), (**e**) are shown in the source data. Data in (**e**) represent means and standard errors

To investigate the adaptive change of cortical activity involved in the use of the ACMC, the task-related modulation depth (MD) of the high-gamma activity of the input electrode was calculated in each trial. MD was defined as the difference of the average rate of occurrence of high-gamma episodes before and after onset for the appearance of the peripheral target (time zero in Fig. 3c). In representative sessions using M1 activity (Fig. 3a), the MD of the input high-gamma activity increased within 20 min (red line in Fig. 3b) along with task improvement (black line in Fig. 3b). High-gamma activity after the onset of target appearance increased substantially during the last 14 trials of the session compared to the initial 14 trials (red line in Fig. 3c). Therefore, electrical stimuli to the paralysed muscles were produced more effectively (thick black line in Fig. 3c), and the wrist position reached the target range more frequently during the last 14 trials (bottom in Fig. 3c). Population analysis of MD in a total of 108 sessions with all cortico-muscle pairs showed that the monkeys could increase MD volitionally. Similar to results for task performance (Fig. 2b), a significant increase in MD occurred within the initial 2 min and plateaued after 6 min (Fig. 3d, one-way ANOVA; *F*_(9,916)_ = 11.46, *P* = 3.79 × 10^−17^. Asterisks represent *P* < 0.05 by one-way ANOVA with Bonferroni’s correction for post hoc multiple comparisons for all times as compared to initial 2 min and last 20 min, respectively. See also Supplementary Fig. 8 for results of each monkey). Moreover, in each case where the input electrodes were located over the M1, PM and S1 areas, the MD of the input high-gamma activity increased over 20 min, suggesting that the monkeys flexibly learned to modulate volitionally input high-gamma activity at various sensorimotor-related cortical sites to adapt to a novel ACMC (Fig. 3e. See also Supplementary Fig. 9, M1: One-way ANOVA; *F*_(2,249)_ = 64.48, *P* = 2.73 × 10^−23^. PM: One-way ANOVA; *F*_(2,51)_ = 21.21, *P* = 1.98 × 10^−7^. S1: One-way ANOVA; *F*_(2,12)_ = 3.89, *P* = 4.98 × 10^−2^). However, monkey M failed to increase MD when the input electrode was in the FEF. This failure may explain why the monkey was unable to improve the task success rate when the input electrode was in the FEF (Fig. 2d).

It is well-known that muscle stimulation induces muscle fatigue. We analysed the evoked torque induced by electrical stimulation to muscles during a long-lasting session of 60 min. The evoked torque showed an apparent decrease over time, indicating muscle fatigue (Supplementary Fig. 10a). However, the monkey could compensate for muscle fatigue by increasing the gamma activity controlling the ACMC in order to maintain wrist torque and task performance (Supplementary Fig. 10b–e). Thus, despite muscle fatigue, the monkey was able to achieve the required wrist torque throughout the extended session.

### Large-scale adaptive change of spatial cortical activity

**Fig. 4:**
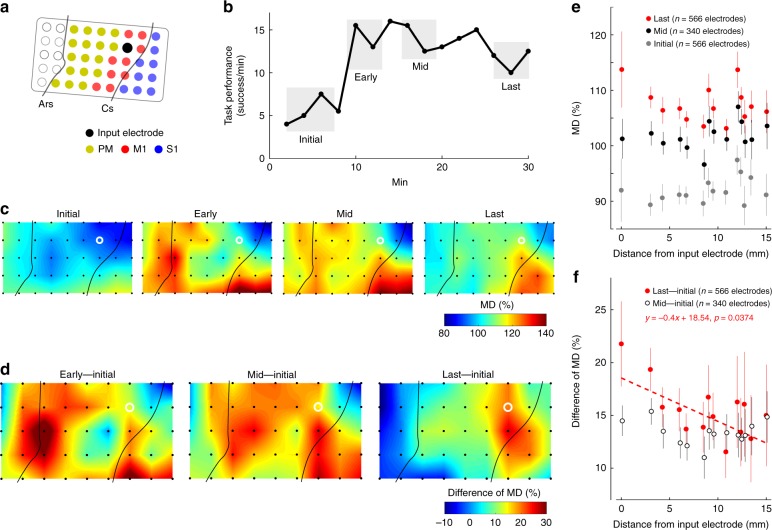
Targeted cortical adaptation during learning with an ACMC. **a** The ACMC input electrode in M1 is shown as a filled black circle. Electrical stimuli were applied to the flexor carpi radialis (FCR) muscle. Data were obtained from Monkey M. **b** Time course of task performance. The data obtained from the time intervals shown in grey shading were used to analyse the MDs of high-gamma activity. **c** Topographic map of MDs during the initial, early, mid, and last phases. **d** Differences in the MD spatial distribution between the initial and early, mid, and last phases. The white circles indicate the input electrode used for the ACMC. Electrodes not used for the ACMC are shown as black dots (**c**–**d**). Similar results were obtained from independent experiments in Monkey TE and M (*n* = 17 sessions, see also Figs. 5 and 6, Supplementary Fig. 11). **e** The relationship between MD and distance from the input electrode in Monkey TE and M (Initial, *n* = 566 electrodes from 17 sessions; Mid, *n* = 340 electrodes from 10 sessions; Last, *n* = 566 electrodes from 17 sessions). The grey, black and red circles represent MD in the initial, mid, and last phases, respectively. The MD continuously increased over initial, mid, and last phases (*P* < 10^−5^ by two-way ANOVA with Bonferroni’s correction for post hoc multiple comparisons). **f** The relationship between the difference of MD versus the distance from the input electrode in Monkey TE and M (Last - Initial, *n* = 566 electrodes from 17 sessions; Mid - Initial, *n* = 340 electrodes from 10 sessions). The black and red circles represent the difference of MD between the initial and mid phases (Mid - Initial) and between the initial and last phases (Last - Initial), respectively. A significant correlation between distance from the input electrode and difference of MD was found only in Last – Initial, shown as a red dotted line (Pearson correlation coefficient; *R* = −0.0875, *P* = 0.0374). Data in (**e**), (**f**) are shown as means and standard errors; detailed statistical results are included in the source data

**Fig. 5:**
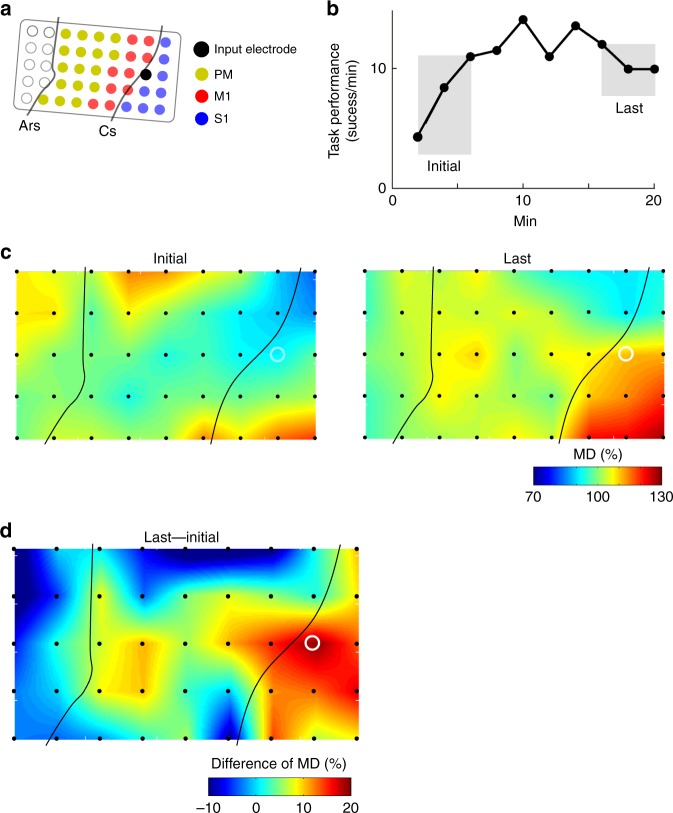
Learning in conjunction with an ACMC in the somatosensory cortex. **a** The ACMC input electrode is represented by the black circle in S1. Electrical stimuli were applied to the flexor carpi ulnaris (FCU) muscle. **b** Time course of task performance over 20 min. The initial and last time intervals used in **c** are indicated in grey. **c** Topographic map of the MD during the initial and last phases. **d** Changes in the spatial distribution of the MD of high-gamma activity in the initial phase compared with the last phase. The white open circle represents the input electrode (**c**–**d**). Similar results were obtained from five independent experiments using different pairs of electrodes from S1 and muscles (*N* = 1 monkey [Monkey M], *n* = 5 sessions, *cf*. Figs. 2c, d and 3e, Supplementary Figs. 6 and 9)

**Fig. 6:**
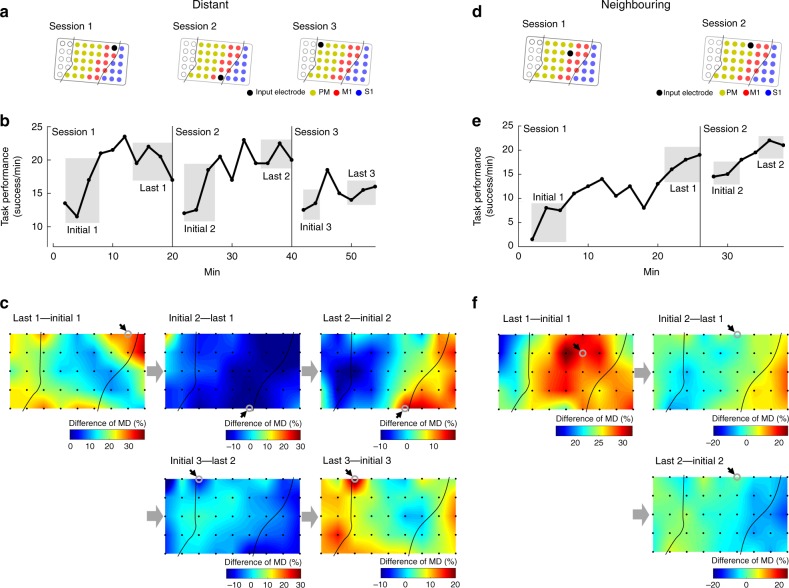
The ACMC induces flexible cortical adaptation. **a**–**c** Three successive sessions using different electrodes in anatomically distant areas. **a** The ACMC input electrode is represented by a black filled circle in each session. The somatotopy of each electrode was evaluated when the monkey was intact. The electrode in M1 was placed in the shoulder area in Session 1, in the digit area in Session 2, and in the shoulder area in Session 3. Electrical stimuli were applied to the FCR muscle in Session 1, the ECR and EDC muscles in Session 2, and the ECU muscle in Session 3. Data were obtained from Monkey M. **b**Time course of task performance in three subsequent ACMC sessions with different input electrodes. The MDs of high-gamma activity were calculated from data obtained during the initial and last phases in each session, indicated in grey. **c** The spatial distribution of MD changes were compared between the initial and last phases in each session (i.e. Last 1 – Initial 1, Initial 2 – Last 1, Last 2 – Initial 2, Initial 3 – Last 2 and Last 3 – Initial 3). The grey open circles with black arrows represent the input electrode. **d**–**f** Two successive sessions using electrodes in nearby areas. **d** The ACMC input electrode, shown as a black filled circle, was in the vicinity of the PM in consecutive sessions. Electrical stimuli were applied to the FCR muscle in Session 1 and the FCU muscle in Session 2. Data were obtained from Monkey M. **e** Time course of task performance in two consecutive sessions with the input electrode in neighbouring sites. MDs were measured during the grey-shaded initial and last phases of each session. **f** Changes in the spatial distribution of MD were compared between the initial and last phases in each session (i.e. Last 1 – Initial 1, Initial 2 – Last 1, and Last 2 – Initial 2). Grey unfilled circles with black arrows represent the input electrode

To further investigate the adaptive changes in cortical activity at extensive cortical sites, the MDs of the high-gamma activity was calculated across all of the recorded electrodes over the FEF, PM, M1 and S1, including the single arbitrarily selected input electrode. Figure 4a–d shows a representative example of the spatial MD map when the ACMC input electrode was located in M1 (Fig. 4a) and the wrist flexor muscle was stimulated. Gradual changes in the spatial MD map were observed over widespread cortical areas as practice with the ACMC progressed (Fig. 4b, c). In the initial phase, MD was distributed relatively uniformly and was low over the recorded area (Initial, Fig. 4c). In the early and middle phases, however, MD increased in various regions around the FEF, PM, and the lateral portion of S1. The MD of the input electrode in M1 increased slightly, but remained lower than that of the other areas (Early and Mid, Fig. 4c). In the last phase, the “hot spots” of MD moved to the S1 area and extended to M1, which included the input electrode, while the hot spot around the FEF and PM in the initial phase shrank gradually over time (Last, Fig. 4c). This tendency was more obvious following calculation of the difference in MD among the initial, early, mid, and last phases (Fig. 4d). This calculation normalised the baseline activity of each recorded site and depicted the change in activity of each stage. The difference in MD between the initial and early phases showed an increase in the activation area in the FEF, PM, and the lateral portion of S1, which were relatively distant from the location of the input electrode (Early–Initial, Fig. 4d). As practice progressed, the difference in MD between the initial and middle phases showed a global increase around the FEF, PM, S1 and M1 (Mid–Initial, Fig. 4d). The difference in MD between the initial and last phases showed that the increase in MD in the FEF, PM and S1 areas diminished, while the MD close to the input electrode in M1 increased, focusing the MD increase to an area around the input electrode (Last–Initial, Fig. 4d). This large-scale adaptive change occurred similarly with the input electrodes over S1 (Fig. 5) and the PM (Session 3, Fig. 6c), suggesting that a large-scale adaptive change of spatial cortical activity among extensive cortical sites facilitates flexible learning with the ACMC. To quantify the spatial scale of modulation that occurred, we analysed the relationship between the MD of each electrode and distance from the input electrode (Fig. 4e), and also between the difference in the MD of each electrode and distance from the input electrode (Fig. 4f). As practice progressed, MD showed a further increase and the largest MD tended to be on the input electrode that was modulated strongly (Fig. 4e, Initial: Two-way ANOVA; [Phase] *F*_(2,1430)_ = 126.95, *P* = 1.77 × 10^−51^, post hoc multiple comparisons with Bonferroni’s correction, *P*_Initial vs Last_ = 1.96 × 10^−51^, *P*_Initial vs Mid_ = 1.12 × 10^−17^, *P*_Mid vs Last_ = 2.10 × 10^−6^). For the change of the spatial map, the difference in MD between the initial and middle phases showed a global and uniform increase irrespective of the distance from the input electrode (Mid–Initial, black in Fig. 4f). In the last phase, the largest difference in MD occurred on the input electrode and was largely increased and decreased on the electrodes surrounding the input electrode (Last–Initial, red in Fig. 4f, Pearson correlation coefficient; *R* = −0.0875, *P* = 0.0374. see also Supplementary Fig. 11 for the data for Monkey TE).

### Flexible targeted adaptation of spatial cortical activity

To examine the flexibility of MD changes, in some sessions, the input electrode was changed suddenly to a novel electrode located in a different cortical area. Fig. 6a–c shows representative task performances and MD maps from three successive sessions using different input electrodes. An electrode in the medial portion of M1 was chosen as the input electrode during session 1, and this was moved to an electrode in the lateral portion of M1 for session 2, and an electrode in the rostromedial portion of the PM for session 3. These sites are located in anatomically distant areas (Fig. 6a). The monkey’s performance improved in each session with each novel electrode (Fig. 6b). Along with the monkey’s improved task performance, the spatial activity pattern changed, as evidenced by the different spatial maps of MD in each session (Fig. 6c). The spatial map of the difference between the initial and late sessions changed drastically in each session, with the strongest modulation concentrated in the area surrounding the input electrode (Last 1–Initial 1, Last 2–Initial 2 and Last 3–Initial 3 in Fig. 6c). To understand the adaptive changes underlying this re-learning strategy, we analysed the spatial map of MD difference in the subsequent session. Comparing MD maps of the late phase of a session and the initial phase of the subsequent session, we observed a global decrease in MD (Initial 2–Last 1 and Initial 3–Last 2 in Fig. 6c). This result indicated that the monkey could reset the learned strategy by decreasing global cortical activity, then, it rapidly and flexibly changed the spatial pattern of high-gamma activity among large-scale cortical areas to a new cortical site.

In another series of sessions, after task performance improved in the first session, we changed the input electrode to a neighbouring electrode (Fig. 6d). The monkey maintained its level of performance and improved further in the second session (Fig. 6e). In this case, the monkey did not globally decrease high gamma activity (Initial 2–Last 1, Fig. 6f), but instead, MD was maintained in the second session (Last 2–Initial 2, Fig. 6f). This suggested that the monkeys were able to utilise the previous learning strategy in second session.

**Fig. 7:**
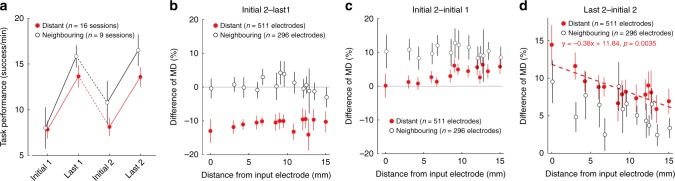
Population data of cortical adaptation to ACMC. **a** Task performance in the initial and last phases of two consecutive ACMC sessions with different input electrodes in Monkey TE and M (Distant electrode [red], *n* = 16 sessions; Neighbouring electrode [black], *n* = 9 sessions). Task performance in Last 1 and Last 2 was significantly higher than in Initial 1 and Initial 2, while task performance after a shift to a neighbouring electrode was significantly higher than after a shift to a distant electrode (*P* < 10^−5^ by two-way ANOVA with Bonferroni’s correction for post hoc multiple comparisons). **b** The relationship between the change in MD (Initial 2 – Last 1) and distance from the input electrode. MD was significantly reduced when the input electrode was shifted to distant sites and unchanged when it was shifted to neighbouring sites. MD changes were not affected by distance from the input electrode. Two-way ANOVA; [Area] *F*_(1)_ = 98.68, *P* = 5.63 × 10^−22^. [Distance] *F*_(13)_ = 0.45, *P* = 0.95, not significant. Post hoc multiple comparisons with Bonferroni’s correction; *P*_Distant vs Neighbouring_ = 5.63 × 10^−22^. **c** The relationship between the difference of MD (Initial 2 – Initial 1) and distance from the input electrode. Difference of MD (Initial 2 – Initial 1) were significantly higher when the input electrode was moved to neighbouring sites compared to when it was moved to distant sites. Two-way ANOVA; [Area] *F*_(1)_ = 34.65, *P* = 5.87 × 10^−9^. Post hoc multiple comparisons with Bonferroni’s correction; *P*_Distant vs Neighbouring_ = 5.87 × 10^−9^. **d** The relationship between difference of MD (Last 2 – Initial 2) and distance from the input electrode. A significant correlation between distance from the input electrode and difference of MD was found only for the distant electrodes, shown by the red dotted line (Pearson correlation coefficient, *R* = −0.129, *P* = 3.49 × 10^−3^). Data represent means and standard errors. Data for **b**–**d** were obtained from Monkey M and TE (Distant (red): *n* = 511 electrodes from 16 sessions; Neighbouring (black): *n* = 296 electrodes from nine sessions). Detailed statistical results for **a**–**d** are shown in the source data

These results were further supported by population analyses (Fig. 7a–d). Task performance was higher when switching input electrodes to neighbouring electrodes than to distant electrodes (Fig. 7a; two-way ANOVA; [Switching] *F*_(1, 92)_ = 4.15, *P* = 4.44 × 10^−2^). When the input electrode was switched to distant sites (Initial 2–Last 1), there was a global and uniform decrease in MD irrespective of distance from the input electrode (Fig. 7b; Two-way ANOVA; [Distance] *F*_(13, 779)_ = 0.45, *P* = 0.95, not significant. [Switching] *F*_(1, 779)_ = 98.68, *P* = 5.63 × 10^−22^, see also Supplementary Fig. 12b, c). This decrease reset MD values close to those observed during the initial phase of session 1 (Fig. 7c, The differences in MD comparing the initial phases of sessions 1 and 2 are close to zero). In contrast, when the input electrode was switched to a neighbouring site, MD values were not reset and session 2 started with the higher MD values observed at the end of session 1 (Fig. 7b, c). When the input electrode was switched to a distant site, the strongest modulation of high gamma activity was localised around the new cortical site controlling stimulation to the muscles (Fig. 7d, Pearson correlation coefficient; *R* = −0.129, *P* = 3.49 × 10^−3^). When the input electrode was switched to a neighbouring site, monkeys maintained the difference of MD from session 1 and exhibited smaller MD changes during the second session (Fig. 7b, c and Supplementary Fig. 12c, d).

## Discussion

This study demonstrates that monkeys with subcortical damage could regain volitional control of a paralysed hand using a closed-loop FES that connects a preserved cortical site with paralysed muscles beyond the lesion. Even in damaged brains, flexible adaptation to a novel ACMC could be achieved through targeted spatial changes in cortical activity in extensive cortical areas. These results suggest that the learning capacity to utilise brain-controlled FES can be harnessed to regain volitional control of a paralysed limb after brain damage.

Among the possible cortical signals available for BMIs, ECoG offers one of the most clinically feasible options, being less-invasive and less technically difficult for electrode implant surgery compared with other recording methods, and having superior long-term stability^23–27^. Furthermore, a high-gamma frequency range recorded using an ECoG array and penetrating electrodes reflects synchronised firing of a neural population^28,29^ and carries information for decoding the activity of multiple muscles^25^, limb movements^26^, and grasping force^27,30^. Here, we established a novel closed-loop FES that was designed to detect the activity pattern of high-gamma oscillations from a single electrode, and converted it in real time to activity-contingent electrical stimulation of muscles. We employed an online template-matching method to detect episodes of high-gamma oscillations from raw oscillatory cortical activity with stimulus artefacts. This high-gamma activity could be utilised as a surrogate for cortical cell activity, allowing monkeys to voluntarily alter electrical stimulation to control paralysed muscles (Fig. 1 and Supplementary Fig. 3). Therefore, oscillatory cortical activity is a suitable control signal for closed-loop FES to the periphery and offers a clinically feasible option.

It has been suggested that controlling a neuroprosthesis requires learning a novel input-output transformation of the closed-loop FES approach^3,8,15^. However, the underlying mechanism involved in learning to control a novel artificial connection has been obscure. Notably, the present study showed that the high-gamma activities of extensive cortical sites, including M1, PM and S1, could be modulated into volitional control of wrist movement via the ACMC (Fig. 3e). A significant correlation between high-gamma neural modulation and volitional wrist movement was confirmed in electrodes over the motor cortex in the intact brain before stroke induction (Supplementary Fig. 13). Therefore, the acquired modulation of high-gamma activity in the motor cortex may be related to the original function of volitional motor execution, suggesting that connecting the motor cortex via the ACMC could result in the restoration of the original descending motor pathways. However, improved task performance in conjunction with the ACMC was similarly achieved irrespective of the original somatotopical organization of M1 (Fig. 6b and Supplementary Fig. 7). This indicates that learning-associated high-gamma neural modulation is flexible and to some degree independent of the original motor function, a result consistent with a previous study demonstrating flexibility in controlling neuronal firing rates in M1 cells^9^.

Task improvement with the ACMC from S1 to muscle (Fig. 5b) suggests that the ACMC could afford the S1 site a “new” function of volitional motor control. It has been shown that monkeys^3,31^ and a human^32^ can learn to modulate volitionally the activity of S1. Recordings during active limb movements have shown that S1 neurons have, in addition to afferent input from peripheral receptors, cortical input that activates them prior to the onset of limb movement^33–36^. This supports a concept important for somatosensory integration during voluntary movements known as the efference copy. Indeed, modulation of M1 and PM activities was observed during S1-controlled ACMC (Fig. 5c, d), suggesting that M1 and the PM might be a source of the efference copy to S1^37^.

Disruption of neuronal networks after neural damage and intensive rehabilitation triggers a large-scale reorganisation at multiple levels of the brain and spinal cord in association with functional improvement. Functional improvement usually takes a few weeks after neural damage has occurred, and the spontaneous reorganisation observed during functional improvement is induced by use-dependent neuroplasticity^18–22,38,39^. Furthermore, longitudinal observations of brain activity demonstrated that adaptive changes in brain activity are associated with functional improvement^21,22,40^. Our results demonstrate that the ACMC induces a large-scale adaptive change in cortical activity that occurs rapidly in extensive cortical areas, including the PM, M1 and S1 (Figs. 4–6). During ACMC sessions, areas of increased activity were first observed in broad cortical areas. Along with task improvement, these areas showed a further increment of their activity and the activated area became more focused on the cortical site that controlled the ACMC (Figs. 4–6). Thus, the improvement in cortical adaptation observed with the ACMC was similar to that observed during recovery after neural damage. However, these adapted-changes occurred substantially faster than those observed during functional improvement after neural damage^21,22^. Moreover, when the input site of the ACMC was changed to a different cortical site, the brain appeared to reset the previous strategy and learned to control the novel site (Fig. 6a–c). Thus, flexible and rapid learning of a novel input-output relationship increases the possible range of both the recognised signals used to control a neuroprosthesis without the need for sophisticated machine learning algorithms, used in previous animal^9,10^ and human^4–6,15^ studies.

Even in a long-lasting session in which the controlling cortical site was switched, the monkeys could maintain motivation and effort throughout the session, as evidenced by the maintenance of task performance (Figs. 6 and 7 and Supplementary Fig. 10). While activity in an extensive area showed a further increase in the last phase of the sessions (Fig. 4e), the activated area became more focused on the cortical site that controlled the ACMC (Fig. 4f). This indicates that trial and errors shaped cortical adaptation in an extensive cortical area, and monkeys could gradually differentiate between cortical sites that showed consistent and inconsistent associations with task success. Association of cortical activity with task success may reinforce activity at the controlling cortical site, while a lack of association with task success may reduce activity at the distant cortical sites. This strategy may be used to reduce total energy costs to maintain task performance.

Although experiments were performed while the monkeys displayed paralysis or severe paresis of the wrist and fingers, all three monkeys showed a gradual improvement of voluntary hand control after the study. It was not clear whether this slight recovery was induced by the ACMC or resulted from natural recovery. Further investigations are needed to clarify if the extended use of an ACMC induces and/or facilitates functional recovery using an animal model of chronic stroke. Moreover, although the present study showed some restoration of the lost voluntary movement at the single wrist joint, increasing the degree of freedom or dexterity to achieve more natural movement is an important challenge^15,16^.

Finally, our results raise a fundamental question regarding which brain regions are responsible for learning in response to the ACMC. In the primate sub-cortical stroke model, most of the descending and ascending pathways are severely damaged. Since the monkeys displayed near complete motor and somatosensory hemiparalysis, somatosensory feedback from the paralysed hand is unlikely to be driving task improvement. Thus volitional control of a paralysed hand via the ACMC may require both physical learning and also abstract learning based on visual or auditory feedback to control the input brain activity^41,42^. It has demonstrated that the striatum play a critical role in reward-based learning^41^ and for the abstract learning of neuroprosthetic control^41^. However, the ability to learn to control the ACMC was well-preserved in our monkeys with unilateral subcortical damage including the striatum. The contralesional striatum might compensate for learning capacity.

In summary, monkeys with subcortical stroke were able to regain volitional control of their paralysed hand via an ACMC, which connected spared cortical sites to muscles using a neural interface. The ACMC induced a large-scale adaptation of spatial cortical activity in a targeted manner. These results indicate that even after subcortical damage, the ACMC imparts a novel function for muscle control to a connected cortical site and triggers cortical adaptation in order to regain the lost volitional movement.

## Methods

### Subjects

The experiments were performed using three male macaque monkeys (two *Macaca fuscata*: Monkey M, 9.0 kg and Monkey TA, 7.5 kg; 1 *Macaca mulatta*; Monkey TE, 9.8 kg). All experimental procedures were performed in accordance with the guidelines of the National Institutes of Health and the Ministry of Education, Culture, Sports, Science, and Technology (MEXT) of Japan, and were approved by the Institutional Animal Care and Use Committee of the National Institutes of Natural Sciences (Approval No. 14A125).

### Surgery

All surgical procedures were performed using sterile techniques while the animal was anesthetized with 1–2% isoflurane. Dexamethasone, atropine, and ampicillin were administered preoperatively; ampicillin and ketoprofen were given post-operatively.

### Cortical implantation surgery

To record oscillatory activities in the brain, we chronically implanted a multichannel ECoG array (Unique Medical Corporation, Tokyo, Japan) in the subdural space in two monkeys (Monkeys TE and M). The ECoG array contained platinum electrodes of 2.1 mm in diameter (1 mm in diameter exposed on a silicone sheet) with an inter-electrode distance of 3 mm. A total of 32 electrodes were implanted in the left hemisphere of Monkey TE, and 45 electrodes were implanted in the left hemisphere of Monkey M, covering the area from the FEF to the primary somatosensory cortex (S1), as shown in Fig. 2c. In Monkey TE, since two of the 32 electrodes that were located on the most caudal part of the array in the left hemisphere were not functional, those electrodes are not shown in Fig. 2c. In all, 16 electrodes were also implanted simultaneously in the right hemisphere, covering M1 and S1. Data from the electrodes in the right hemisphere were not included in the present study. The reference and ground electrodes were placed in the subdural space. In Monkey TA, a moveable tungsten microwire array comprising 20 electrodes^43^ was chronically implanted within the hand area in M1. Electrical cables leading from the electrode array were affixed to connectors anchored to the skull with titanium screws. The cortical electrodes and head-post chamber were anchored with screws and acrylic cement.

### Surgery for muscle stimulation

To deliver electrical stimulation to the muscle, bipolar stimulating wires were chronically implanted (Monkeys M and TA). The stimulus electrodes were surgically implanted in the arm, wrist, and hand muscles in a total of 15 muscles in Monkey M and 12 muscles in Monkey TA, as identified by their anatomical features and by movements evoked by trains of low-intensity stimulation. Bipolar, multi-stranded stainless steel wires (AS633; Cooner Wire, Chatsworth, CA, USA) were sutured into each muscle, and the wires were routed subcutaneously to a connector on the animal’s head. In Monkey M, electrodes were implanted in the shoulder muscles (deltoid, del), elbow muscles (triceps, tri; biceps brachii, BB), wrist flexor muscles (carpi radialis, FCR; palmaris longus, PL; pronator teres, PT; digitorum sublimis, FDS; carpi ulnaris, FCU), wrist extensor muscles (carpi ulnaris, ECU; digitorum communis, EDC; carpi radialis, ECR; brachioradialis, BR), and digit muscles (digitorum 4 and 5, ED45; abductor pollicis longus, APL; adductor pollicis, ADP). In Monkey TA, electrodes were implanted in the elbow muscles (Tri, BB), wrist flexor muscles (FCR, PL, FDS, FCU), wrist extensor muscles (ECU, EDC, ECR, BR), and digit muscles (extensor digitorum 2 and 3, ED23; first dorsal interosseous, FDI). In Monkey TE, the electrical stimuli were delivered transcutaneously to arbitrarily selected wrist synergistic muscles so that surgery was not performed.

### Surgery to generate a primate sub-cortical stroke model

A stroke model was generated by occluding the lenticulostriate arteries (LSA) in Monkeys TA and M. In Monkey TE, we occluded both the LSA and the anterior choroidal artery (AChA) (Supplementary Fig. 1). In order to expose the origin of the LSA and AChA, which arise just proximal to the top of the internal carotid artery (ICA), we used a left frontotemporal approach. The animals were fixed to a stereotaxic frame in a supine position with their head rotated 15° towards the right side. After removal of the bone flap, the dura was opened. The left frontal lobe was elevated to expose the optic chiasma and ICA. The ICA was traced proximally to identify the origin of the AChA, which arises from the posterior wall of the ICA and courses along the optic tract. The AChA was coagulated and transected just distal to the origin of the AChA. The ICA was traced distally to identify the horizontal portion of the middle cerebral artery (MCA). The arachnoid and trabecula around the MCA were dissected out to expose the origin of the LSA, which arises anterosuperiorly from the MCA. The LSA was coagulated and transected. The dura was sealed, and a cranioplasty with polymethyl methacrylate was performed.

### Data collection

In Monkeys TE and M, the ECoG electrode arrays were connected to a multi-channel amplifier. A total of 45 (Monkey M) or 48 (Monkey TE) ECoG signals were recorded simultaneously with a Cerebus™ data acquisition system (Blackrock Microsystems, Salt Lake City, UT, USA) at a sampling rate of 10 kHz. In Monkey TA, 16 local field potential signals were recorded simultaneously at a sampling rate of 3.5 kHz with a CED 1401 data acquisition system (CED, Cambridge, UK). Then, the ECoG or local field potential signals were filtered with second-order Butterworth filters with corners at 0.3 Hz and 1 kHz, and a notch filter was used to remove the noise caused by the AC components of the power supply (60 Hz).

### Artificial cortico-muscular connection

To achieve an Artificial cortico-muscular connection (ACMC) that sent voluntary commands to the paralysed muscles and bypassed the stroke, cortical oscillatory activity was converted into stimulus pulses. The ACMC was accomplished using a computer interface that was designed to detect high-gamma neural oscillations (80–120 Hz) specifically using a template-matching algorithm and converted in real time to activity-contingent electrical stimuli dependent on the rate of occurrence.

Before the ACMC session, we set a 1–3-min period as a baseline epoch in which the monkey viewed a blank screen; this allowed us to use a spike-sorting device based on a template-matching algorithm (MSD; Alpha Omega Engineering, GA, USA) to identify the particular waveforms of filtered high-gamma oscillations. High-gamma waveforms with a relatively high amplitude used for the ACMC input signal were selected such that they could be detected reliably from stimulus artefacts (Supplementary Fig. 2). The moving averaged rate of occurrence (250-ms time window) of the selected high-gamma episodes had a proportional relationship with both the stimulation current and frequency; thus, the monkeys could voluntarily co-modulate the current and frequency of the electrical stimuli dependent on the single source of the occurrence rate of the high-gamma events. If the average occurrence rate of the selected high-gamma episodes (*X* [Hz]) was above the stimulus threshold (*X*_th_ [Hz]), the stimulus frequency (*f* [Hz]) and current (*I* [mA]) were modulated by the following equations^12,13^:

1 $$f = f_0 + \frac{{f_{\mathrm{g}}}}{{X_{{\mathrm{th}}}}} \cdot X,\left( {f_0 \le f \le f_{{\mathrm{Max}}}} \right)$$

(*f*_0_ = initial stimulus frequency when *X* [Hz] was above *X*_*th*_ [Hz], *f*_g_ = gain of the stimulus frequency, *f*_Max_ = maximum stimulus frequency [Hz])

2 $$I = I_0 + \frac{{I_{\mathrm{g}}}}{{X_{{\mathrm{th}}}}} \cdot X,\left( {I_0 \le I \le I_{{\mathrm{Max}}}} \right)$$

(*I*_0_ = initial stimulus current, *I*_g_ = gain of the stimulus current, *I*_Max_ = maximum stimulus current [mA]).

Each stimulus parameter was also determined in the baseline epoch in each session using the following criteria: X_th_, higher than the average occurrence rate of the detected high-gamma episodes during the baseline epoch; ***f***_0_, the stimulus frequency that could induce a smooth movement trajectory; ***f***_g_ and ***I***_g_, the gains of stimulus frequency and intensity that allowed ***f***_Max_ and ***I***_Max_ to be reached while the participant held the peripheral target, respectively; ***f***_Max_ and ***I***_Max_, the maximum stimulus frequency and intensity that allowed tetanic contraction while the participant held at the peripheral target, respectively. Across the monkeys, these parameters ranged as follows: *X*_th_, 6.5–10 Hz; ***f***_0_, 5–10 Hz; ***f***_g_, 0.3–0.5 Hz; *f*_Max_, 20–35 Hz; ***I***_0_, 1.5–2.2 mA; ***I***_g_, 0.05–0.08 mA; and *I*_Max_, 3.4–4.5 mA. The initial stimulus current (***I***_0_), initial stimulus frequency (***f***_0_), and maximum stimulus current (*I*_Max_) were sometimes adjusted to reduce muscle fatigue and maintain a consistent relationship between wrist torque and the rate of occurrence of selected high-gamma episodes. However, improvements in the rate of target success were not due to changing the task difficulty or stimulus gain, as the target parameters and stimulus gains were not significantly different. Electrical stimulation was delivered to a pair of arbitrarily selected synergist muscles through the stimulator (Bp Isolator; FHC, Bowdoin, ME, USA). In Monkey TE, the electrical stimuli were applied transcutaneously to the arbitrarily selected wrist synergistic muscles, which were confirmed by visual inspection. After confirming the direction of the evoked movements, the target was set in the direction that corresponded to the direction of the evoked movement or torque.

### Behavioural task

Each monkey controlled the position of a cursor on a video monitor with isometric flexion and extension of wrist torque (torque-tracking task) or position (position-tracking task), and acquired targets displayed on the screen. The monkey was required to maintain torque within each target for 0.3–1.8 s to receive a juice reward. The targets remained on the screen until the hold criterion was met, followed by presentation of the next target, either immediately or after a variable reward period. The monkeys participated in a total of 72 torque-matching task sessions with the ACMC (Monkey TE, 11 sessions; Monkey TA, 28 sessions; Monkey M: 33 sessions) and 36 position-matching tasks with the ACMC (Monkey TA, 32 sessions; Monkey M, four sessions). Some sessions included a three-graded torque-matching task (Monkey TA, 19 sessions; Monkey M, 12 sessions) or a three-graded position-tracking task (Monkey TA, 17 sessions). The duration of task performance was 20.2 ± 8.5 min across the total of 108 sessions. Task performance was calculated by the average number of successfully acquired targets across the total number of sessions per every 2 min.

### MD of the input high-gamma activity

As was described briefly in the Results section, we first calculated the MD of the rate of occurrence of the arbitrarily detected high-gamma episodes extracted from the input electrode. MD was defined as the difference of the average instantaneous occurrence rate of high-gamma episodes before and after peripheral target appearance. The time range before and after peripheral target appearance for the analysis was adjusted from 0.5 to 1.5 s depending on the holding time of the peripheral target in each session. Then, we compared the averaged MD across the total number of sessions per every 2 min. To elucidate further the adaptive change of cortical activities over extensive cortical areas, we quantified the MD of input-related high-gamma activity in all of the recorded electrodes, including the input and non-input electrodes. In this study, high-gamma neural waveforms were selected arbitrarily as the input signal that controlled the ACMC. Therefore, we hypothesised that the calculation of the MD of the high-gamma power spectrum in each electrode should reflect learning-related neural changes in widespread cortical areas. The MD of the power spectrum density was extracted from the high-gamma neural oscillations observed 1.5–2.0 s prior to and 2.0–3.5 s after peripheral target appearance.

Consequently, artefacts were induced by the electrical pulses delivered to the muscles and were present in the recorded neural signals. To minimise the effect of artefact contamination, the raw data from 2 ms before to 30 ms after aligned with stimulus timing were removed, and then the remaining raw data were analysed. The time period chosen for elimination was determined based on visual confirmation of the data calculated from the stimulus-triggered average of the raw ECoG data. We omitted the data if either time window included a time point before or after target appearance within 1.024 s. For the power spectral analysis, we used a discrete Fourier transform and its derivations calculated in accordance with the method of Halliday et al.^44^. The raw 10-kHz ECoG data were downsampled to 1 kHz. ECoG power spectral density was calculated by dividing a waveform signal into at least one section of the same duration of 1,024 ms (1,024 data points without overlap). Each section was windowed (Hanning window), and the magnitude of the discrete Fourier transform of each section was averaged to form the power spectrum density, yielding a frequency resolution of 0.987 Hz. Then, the power spectrum in the frequency band of interest (80–120 Hz) was summed, and the ratio before and after target appearance was computed. Data analysis was performed with MATLAB (The MathWorks Inc., MA, USA).

### Statistical analysis

To determine statistically significant differences in task performance among the initial ACMC phase, the last ACMC phase, and the catch trials in the three monkeys (Fig. 2a) and in each monkey (Supplementary Fig. 4a), one-way factorial ANOVA with Bonferroni’s correction for post hoc multiple comparisons was performed. To determine statistically significant differences in task performance in every 2 min in the three monkeys (Fig. 2b) and in each monkey (Supplementary Fig. 4b), one-way ANOVA with repeated measures with Bonferroni’s correction for post hoc multiple comparisons was conducted. To determine statistically significant differences in task performance in every 2 min among the different input electrodes over the PM, M1, and S1, two-way ANOVA with repeated measures with Bonferroni’s correction for post hoc multiple comparisons was conducted (Fig. 2d). To determine statistically significant differences in task performance among the initial ACMC phase, the last ACMC phase, and the catch trials between the position- and torque-tracking tasks (Supplementary Fig. 5a) and between the two- and three-graded tracking tasks (Supplementary Fig. 5c), two-way factorial ANOVA with Bonferroni’s correction for post hoc multiple comparisons was performed. To determine statistically significant differences in task performance in every 2 min between the position- and torque-tracking tasks (Supplementary Fig. 5b) and between the two- and three-graded tracking tasks (Supplementary Fig. 5d), two-way ANOVA with repeated measures with Bonferroni’s correction for post hoc multiple comparisons was performed. To determine significant differences in task performance between the initial 2 min, last 2 min, and peak 2 min for each input electrode over the PM, M1, and S1, two-way factorial ANOVA with Bonferroni’s correction for post hoc multiple comparisons was performed (Supplementary Fig. 6). To determine statistically significant differences in MD in every 2 min among the different input electrodes over the PM, M1 and S1, two-way ANOVA with repeated measures with Bonferroni’s correction for post hoc multiple comparisons was conducted (Fig. 3d). To determine statistically significant differences in MD in every 2 min among the different input electrodes over the PM, M1, and S1, two-way ANOVA with repeated measures with Bonferroni’s correction for post hoc multiple comparisons was conducted (Fig. 3e).

To determine statistically significant differences in MD in every distances from the input electrode among initial ACMC phase, mid ACMC phase, and last ACMC phase, two-way ANOVA with repeated measures with Bonferroni’s correction for post hoc multiple comparisons was conducted (Fig. 4e). To determine statistically significant differences in MD in every distances from the input electrode among initial ACMC phase, and last ACMC phase in case that the input electrodes were located over the S1, two-way ANOVA with repeated measures with Bonferroni’s correction for post hoc multiple comparisons was conducted (Fig. 5e). To determine statistically significant differences in MD difference (Last 1 – Initial 2 in Fig. 7b; Initial 2 – Initial 1 in Fig. 7c) in every distances from the input electrode when the input electrodes in session 2 were located in distant or neighbouring sites, two-way ANOVA with repeated measures with Bonferroni’s correction for post hoc multiple comparisons was conducted. Pearson correlation coefficients were computed between the difference of MD and distance from the input electrode (Last – Mid and Last – Initial in Fig. 4f; Last – Initial in Fig. 5f; Last 2 – Initial 2 in Fig. 7d). Statistical analysis was performed with MATLAB (The MathWorks Inc., MA, USA) statistical tool box.

### Confirmation of lesion extent

To identify the lesion area induced by subcortical stroke, we used magnetic resonance imaging (MRI) or histological sections.

Under deep anaesthesia (pentobarbital 20 mg/kg, i.v.), we performed MRI on Monkey TE using a 3T scanner (Allegra 3T; Siemens Healthcare) with a standard head coil. T2-weighted MRI was performed using a two-dimensional turbo spine echo sequence (0.5 × 0.5 mm, 40 slices, slice thickness = 1.0 mm, TR/TE = 7880/19 ms, number of average = 4). The hyperintense area in T2-weighted images was defined as the extent of the lesion (Supplementary Fig. 1a).

At the end of the experiments in Monkeys M and TA, they were deeply anesthetized with an overdose of sodium pentobarbital (50–75 mg/kg, i.v.) and perfused transcardially with 0.1 M PBS (pH 7.4), followed by 4% paraformaldehyde in 0.1 M phosphate buffer (pH 7.4). The brain was removed immediately and immersed successively in 10, 20, and 30% sucrose solution of 0.1 M PBS (pH 7.3). Then, they were cut serially into 50-μm-thick coronal sections on a freezing microtome. All sections were processed for Nissl-staining with 1% cresyl violet. Photomicrographs of the subcortical lesion were captured. The extent of the lesion was defined by the area of gliosis (Supplementary Fig. 1b). Since we failed to perfuse the brain of Monkey TA, we cannot show the extent of its lesion.

### Reporting summary

Further information on research design is available in the Nature Research Reporting Summary linked to this article.

## Supplementary information

Supplementary Information

Peer Review File

Reporting Summary

Source Data

## Data Availability

Source Data for all Figures in main text have been provided with the paper. Original data that supports the findings are available from the corresponding author upon reasonable request.

## Code availability

MATLAB (Math Works Inc., MA, USA) codes used for data analysis are available from the corresponding author upon reasonable request.
