# Supplementary material for: Bypassing stroke-damaged neural pathways via a neural interface induces targeted cortical adaptation
Source: Nat Commun. 2019 Oct 16;10:4699. doi: 10.1038/s41467-019-12647-y (PMC6796004; doi:10.1038/s41467-019-12647-y)
Supplement: Supplementary file 1 — Supplementary Information [file 41467_2019_12647_MOESM1_ESM.pdf]

Supplementary information for

Bypassing stroke-damaged neural pathways via a neural interface induces targeted cortical adaptation

Kenji KATO[1,2,3], Masahiro SAWADA[1,4], Yukio NISHIMURA[1,2,5,6,7]

1. Department of Developmental Physiology, National Institute for Physiological Sciences, 38 Nishigonaka, Myodaiji, Okazaki, 444-8585, Aichi, Japan
2. Department of Physiological Sciences, School of Life Science, The Graduate University for Advanced Studies, SOKENDAI, Shonan Village, Hayama, 240-0193, Kanagawa, Japan
3. Japan Society for the Promotion of Science, Kojimachi Business Center Building, 5-3-1 Kojimachi, Chiyoda-ku, 102-0083, Tokyo, Japan
4. Department of Neurosurgery, Graduate School of Kyoto University, 54 Shogoin-kawaharacho, Sakyo-ku, 606-8507, Kyoto, Japan
5. Neural Prosthetics Project, Department of Dementia and Higher Brain Function, Tokyo Metropolitan Institute of Medical Science, 2-1-6, Kamikitazawa, Setagaya, 158-8506, Tokyo, Japan
6. Department of Neuroscience, Graduate School of Medicine and Faculty of Medicine, Kyoto University, Yoshida-Konoe, Sakyo, Kyoto 606-8501, JAPAN
7. Precursory Research for Embryonic Science and Technology, Japan Science and Technology Agency, Sanban-tyo, Chiyoda, 102-0076, Tokyo, Japan

Corresponding author:

Yukio Nishimura, Ph. D.

Neural Prosthetics Project, Department of Dementia and Higher Brain Function, Tokyo Metropolitan Institute of Medical Science

Address: 2-1-6, Kamikitazawa, Setagaya, 158-8506, Tokyo, Japan

Tel: 81-3-6834-2373

E-mail: nishimura-yk@igakuken.or.jp

Supplementary Figures

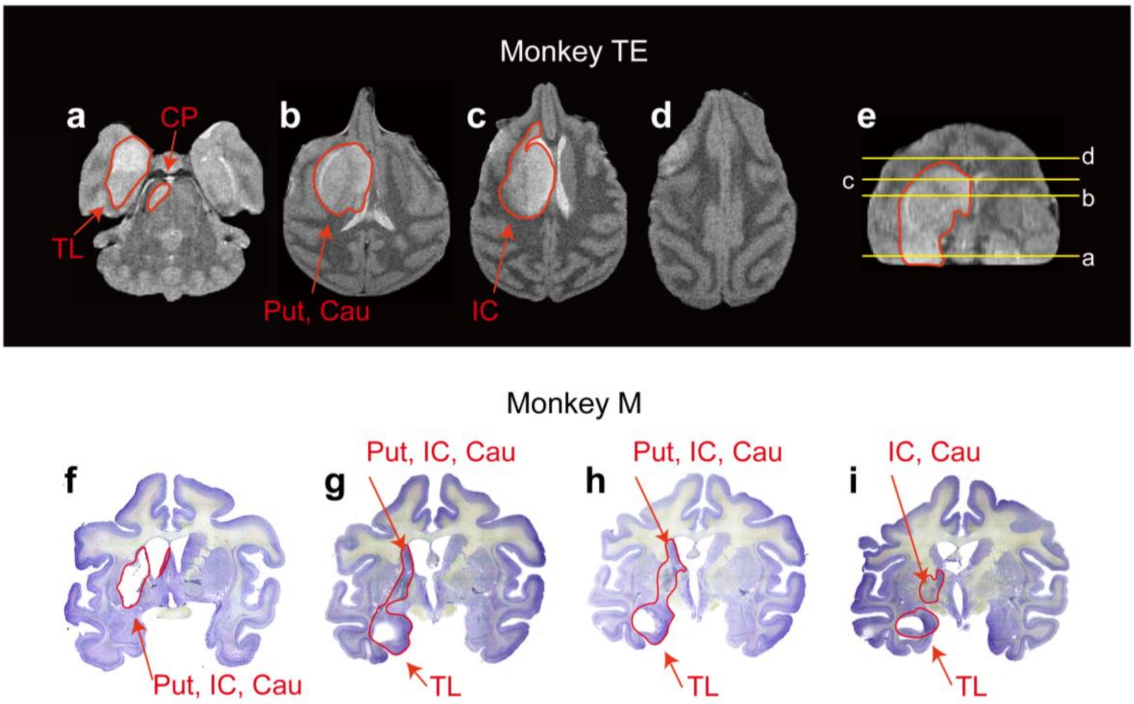

Supplementary Figure 1. Lesion extent in a primate sub-cortical stroke model. a-e: Transaxial T2-weighted magnetic resonance images showing the extent of the lesion in Monkey TE. A stroke was generated by occluding the lenticulostriate arteries and anterior choroidal artery. The red lines represent the lesion area, which included the temporal lobe (TL in **a**), cerebral peduncle (CP in **a**), putamen (Put in **b**), caudate (Cau in **b**), and internal capsule (IC in **c**). The yellow lines in **e** indicate the transaxial position of the coronal sections shown in **a–d**. **f–i**: Nissl-stained sections show the extent of the lesion in Monkey M. A stroke was generated by occluding the lenticulostriate arteries. The red lines represent the lesion area, which included the Put (in **f–h**), IC (in **f–i**), Cau (in **f–h**), and TL (in **g–i**).

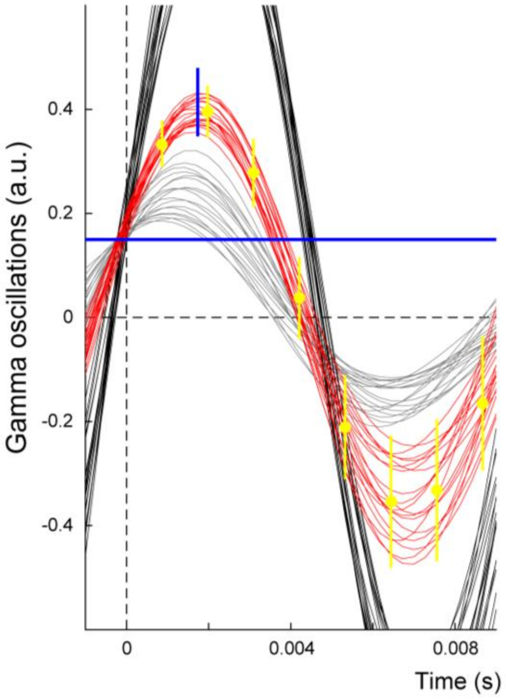

Supplementary Figure 2. Detection of high-gamma episodes. An arbitrary one-cycle high-gamma waveform was detected using a template-matching algorithm to identify the particular shape and amplitude of the waveforms. Waveforms with a relatively high amplitude which could be differentiated reliably from stimulus artifacts were selected for the ACMC input signal. Red traces indicate the high-gamma waveforms detected using the template-matching method, with thresholds represented as blue lines. The yellow lines represent the template for high-gamma waveform detection. Black lines indicate stimulus artifacts that go off scale.

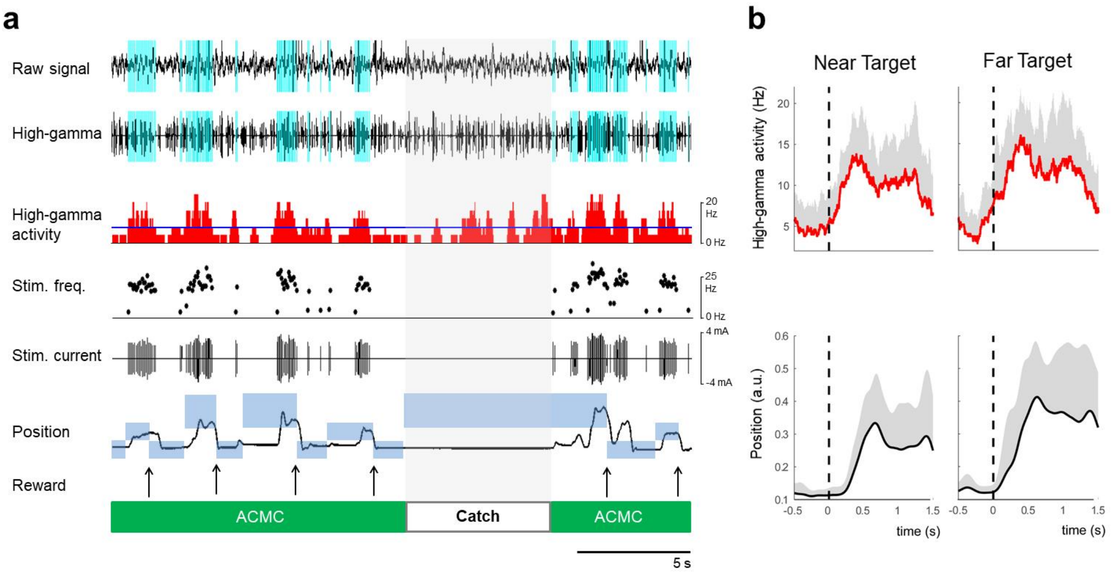

Supplementary Figure 3. Volitional control of a paralysed hand during a three-graded position-tracking task with the artificial cortico-muscular connection (ACMC). a: A representative example of six successful trials in a three-graded position-tracking task with the ACMC (green) and one catch trial (grey shading) in Monkey TA. Monkey TA acquired three randomly presented different levels of wrist positions using high-gamma activity to grade muscle stimulation delivered to the flexor carpi ulnaris (FCU) and flexor carpi radialis (FCR) muscles. Raw oscillatory cortical activity (1st row) was recorded from a microelectrode in the hand area of the primary motor cortex (M1), and filtered for high-gamma frequency bands (2nd row). The blue areas in the 1st and 2nd rows indicate the timing of electrical stimulation. An arbitrary high-gamma neural oscillation was selected as the input signal to control the ACMC. The frequencies of the detected high-gamma waveforms were smoothed in 250-ms bins (3rd row). FCU and FCR muscles were stimulated with a frequency (4th row) and current (5th row) proportional to the smoothed high-gamma activity above a stimulation threshold, shown as the blue horizontal line in the 3rd row. In the 6th row, blue rectangles indicate three different wrist positions. Arrows at the bottom indicate successful task completion and reward times. When the ACMC was on (green), the monkey was able to modulate high-gamma activity volitionally to control the stimulation to the paralysed muscles using the ACMC; thus, the monkey could repeatedly acquire the three-graded targets. During the catch trial (grey shading) when the ACMC was off, the monkey made attempts to move its wrist, as evidenced by the increase in high-gamma activity above the threshold, but failed to acquire the target. **b**: Representative data of the averaged high-gamma activity and hand

position over the last 15 trials for near (left) and far (right) targets with the ACMC in Monkey TA. The plots are aligned to the time onset of target appearance, shown as vertical dotted lines. Gray area represent standard error. See also population data in Supplementary Fig. 5c and 5d (**a**-**b**).

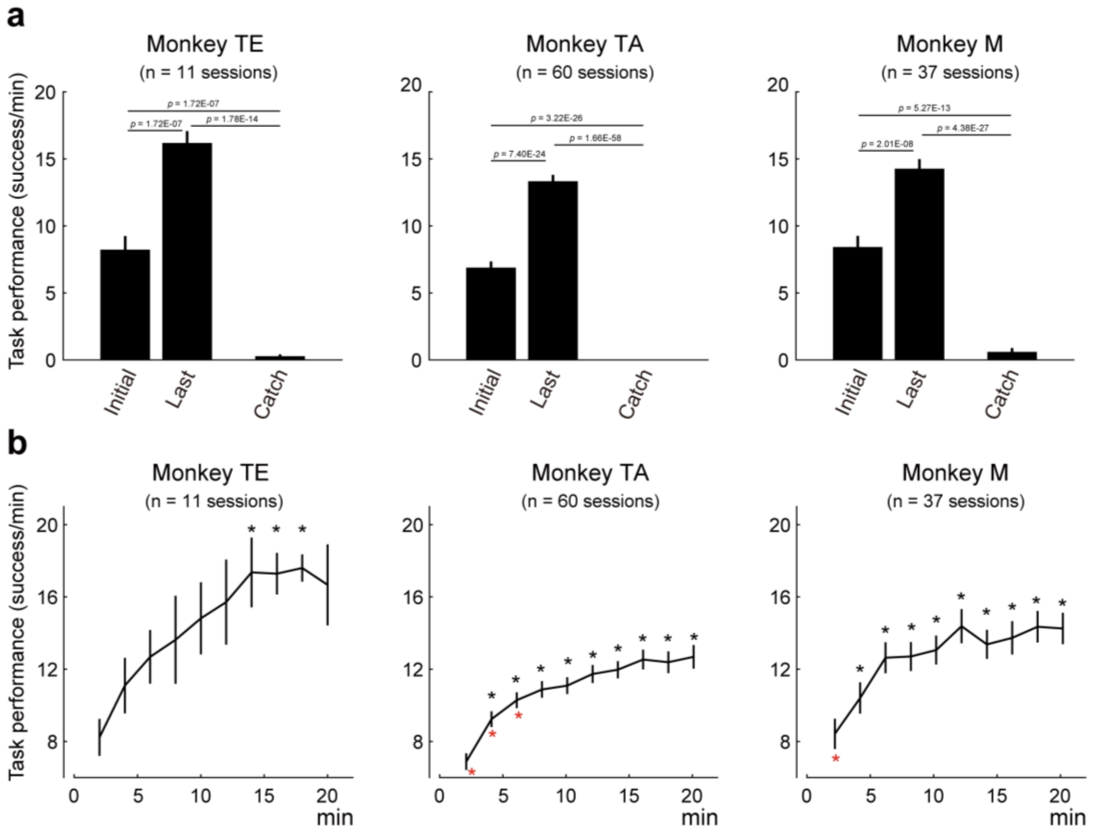

Supplementary Figure 4. Task performance in the three monkeys. a: Average task performance during the initial 2 min and last 2 min of sessions, and during catch trials is shown for each monkey. One-way ANOVA; Monkey TE (n = 11 sessions): $F_{(2, 30)} = 102.61$ and $P = 3.84 \times 10^{-14}$, Monkey TA (n = 60 sessions): $F_{(2, 177)} = 302.47$ and $P = 7.94 \times 10^{-58}$, Monkey M (n = 37 sessions): $F_{(2, 108)} = 109.03$ and $P = 1.22 \times 10^{-26}$. Data are shown as mean values with standard errors. **b:** The time course of task success/time using the ACMC for each monkey. Task performance improved over time for all monkeys. One-way ANOVA; Monkey TE (n = 11 sessions): $F_{(9, 68)} = 3.38$ and $P = 1.78 \times 10^{-3}$, Monkey TA (n = 60 sessions) $F_{(9, 520)} = 13.70$ and $P = 7.67 \times 10^{-20}$, Monkey M (n = 37 sessions): $F_{(9, 308)} = 5.17$ and $P = 1.52 \times 10^{-6}$. Black asterisks indicate significant differences ($P < 0.05$ by one-way ANOVA with Bonferroni's correction for *Post hoc* multiple comparisons) compared to the initial 2 min bin, and red asterisks indicate significant differences compared to the last 2 min bin. Cortical signals were recorded by ECoG array in monkey M and TE, and microelectorodes in Monkey TA respectively. Data are shown as mean values with standard errors. Detailed statistical results for **a** and **b** are shown in the source data.

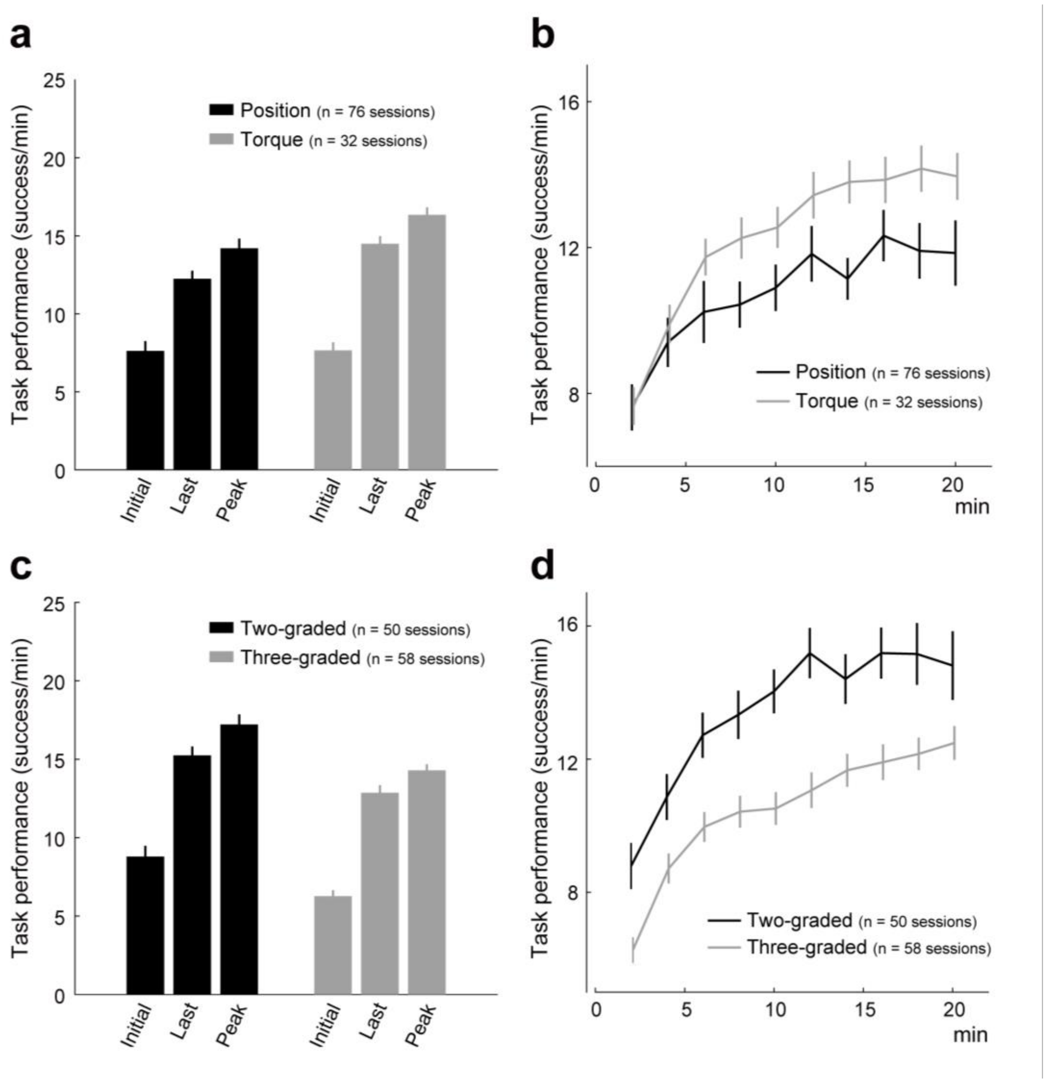

Supplementary Figure 5. Comparison of task performance in relation to task difficulty. a: Average task performance of the position- and torque-tracking tasks. Torque-tracking: N = 3 monkeys, n = 76 sessions. Position-tracking: N = 2 monkeys [Monkey TA and M], n = 32 sessions. Task performance of the torque-tracking task was significantly higher than that of the position-tracking task. Two-way ANOVA; [Task] $F_{(1, 318)} = 7.53$, $P = 6.42 \times 10^{-3}$, [Phase] $F_{(2, 318)} = 93.71$, $P = 1.01 \times 10^{-32}$. **b:** Time course of average performance in position- and torque-tracking tasks. Performance improved over time for both tasks. Performance in the torque-tracking task was significantly higher than performance in the position-tracking task. Two-way ANOVA; [Time] $F_{(9, 906)} = 14.61$, $P = 2.87 \times 10^{-22}$, [Task] $F_{(1, 906)} = 23.73$, $P = 1.30 \times 10^{-6}$. **c:** Average task performance in two- and three-graded tracking tasks. Two-graded: N = 3 monkeys, n = 50 sessions. Three-graded: N = 2 monkeys [Monkey TA and M], n = 58 sessions. Performance in the

two-graded task was significantly higher than that of the three-graded task. Two-way ANOVA; [Task] $F_{(1, 318)} = 40.57$, $P = 6.44 \times 10^{-10}$, [Phase] $F_{(2, 318)} = 135.72$, $P = 2.43 \times 10^{-43}$. **d**: Time course of task performance in the two- and three-graded tracking tasks. Performance improved over time for both tasks, and performance in the two-graded task was significantly higher than that of the three-graded task. Two-way ANOVA; [Time] $F_{(9, 906)} = 21.92$, $P = 7.54 \times 10^{-34}$, [Task] $F_{(1, 906)} = 119.59$, $P = 3.12 \times 10^{-26}$. Cortical signals were recorded by ECoG array in monkey M and TE, and microelectorodes in Monkey TA respectively. Detailed statistical results for (**a-d**) are shown in the source data. Data in all panels are shown as means with standard errors.

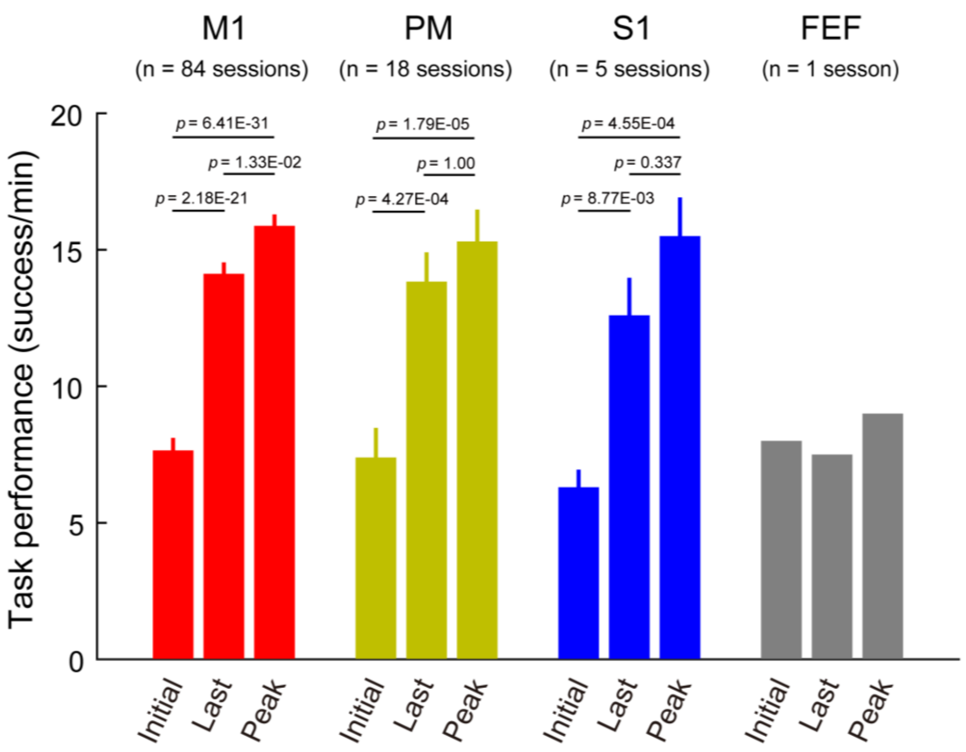

Supplementary Figure 6. Task performance at various cortical sites. Task performance with the input electrode over the M1 (N = 3 monkeys, n = 84 sessions), PM (N = 2 monkeys [Monkey TE and M], n = 18 sessions), S1 (N = 1 monkey [Monkey M], n = 5 sessions), and FEF (N = 1 monkey [Monkey M], n = 1 session) during the initial, last, and peak phases in the three monkeys. In M1, PM, and S1, significant increases in task performance in the last and peak phases compared to that in initial phase were found (One-way ANOVA; $F_{(2, 249)} = 100.49$, $P = 1.01 \times 10^{-32}$ in M1, $F_{(2, 51)} = 14.44$, $P = 1.07 \times 10^{-5}$ in PM, and $F_{(2, 12)} = 15.44$, $P = 4.81 \times 10^{-4}$). Cortical signals were recorded by ECoG array in monkey M and TE, and microelectorodes in Monkey TA respectively. Detailed statistical results are shown in the source data. Data represent mean values and standard errors.

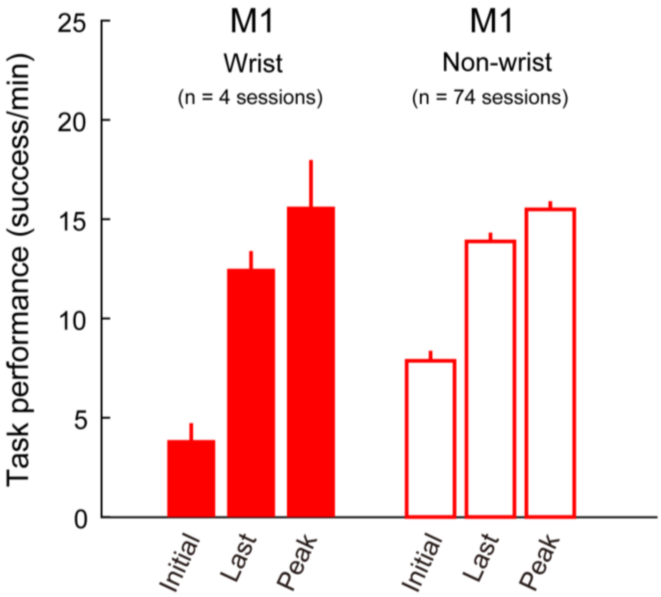

Supplementary Figure 7. Task performance at different somatotopic sites in M1. Wrist site (N = 1 monkey [Monkey M], n = 4 sessions), non-wrist site (N = 2 monkeys [Monkey TA and M], n = 74 sessions). Task performance were identical irrespective of the original somatotopy of M1 before stroke (Two-way ANOVA; [Somatotopy] $F_{(1, 228)}$ = 2.32, P = 0.13, not significant). Cortical signals were recorded by ECoG array in monkey M, and microelectorodes in Monkey TA respectively. See source data for detailed statistical analyses. Data represent means and standard errors.

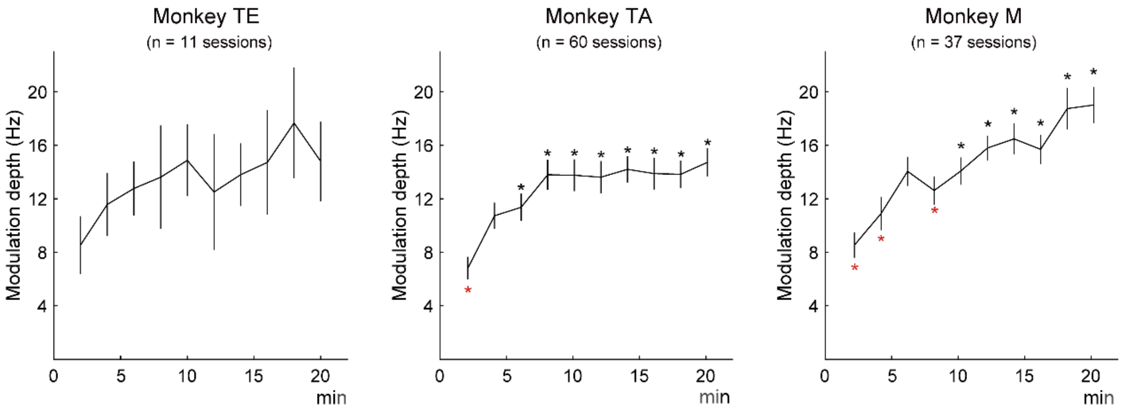

Supplementary Figure 8. Changes in the modulation depth (MD) of high-gamma signals. The MD of input high-gamma signals in each monkey increased similarly over 20 min. One-way ANOVA; Monkey TE (n = 11 sessions): $F_{(9, 68)} = 0.647$ and $P = 0.753$, Monkey TA (n = 60 sessions): $F_{(9, 520)} = 5.48$ and $P = 3.32 \times 10^{-7}$, Monkey M (n = 37 sessions): $F_{(9, 308)} = 8.07$ and $P = 9.39 \times 10^{-11}$. Black and red asterisks indicate $P < 0.05$ (one-way ANOVA with Bonferroni's correction for *Post hoc* multiple comparisons) compared to the initial 2 min and the final 2 min, respectively. Cortical signals were recorded by ECoG array in monkey M and TE, and microelectorodes in Monkey TA respectively. Detailed statistical results are shown in the source data. Data represent mean values and standard errors.

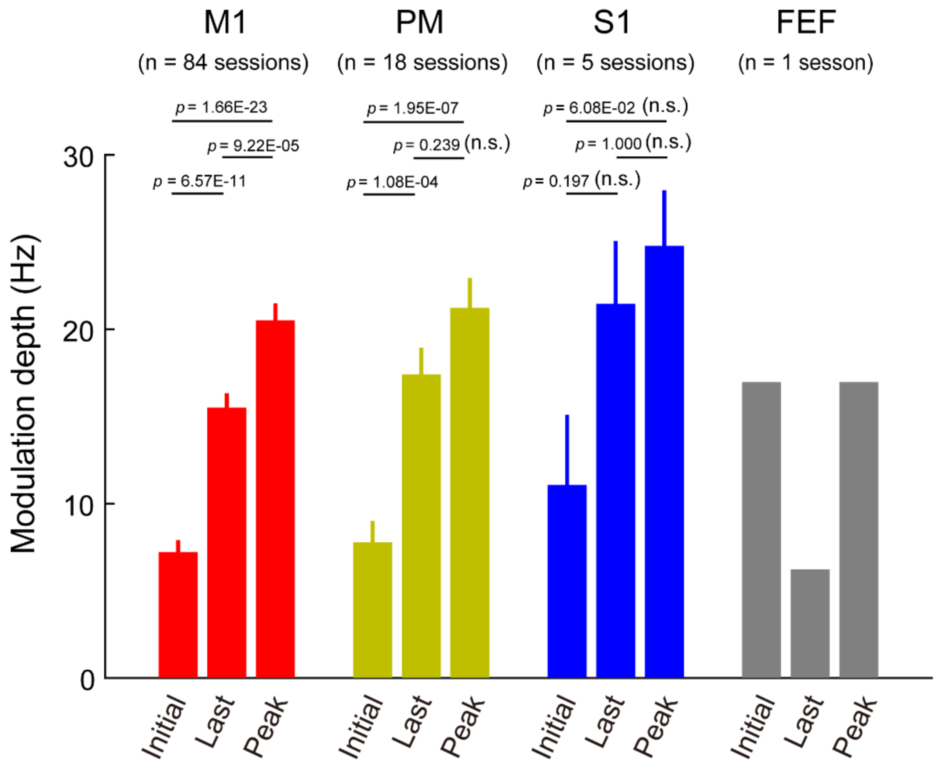

Supplementary Figure 9. Modulation depth at various cortical sites. Modulation depth with the input electrode over the M1 (N = 3 monkeys, n = 84 sessions), PM (N = 2 monkeys [Monkey TE and M], n = 18 sessions), S1 (N = 1 monkey [Monkey M], n = 5 sessions), and FEF (N = 1 monkey [Monkey M], n = 1 session) during initial, last, and peak phases. Significant increases in task performance in the last and peak phases were found in M1, PM, and S1 (One-way ANOVA; $F_{(2, 249)}$ = 64.48, P = 2.73 × 10^{-23} in M1, $F_{(2, 51)}$ =21.21, P = 1.98 × 10^{-7} in PM, and $F_{(2, 12)}$ =3.89, P = 4.98 × 10^{-2} in S1). Cortical signals were recorded by ECoG array in monkey M and TE, and microelectorodes in Monkey TA respectively. Detailed statistical results are shown in the source data. Data represent mean values and standard errors.

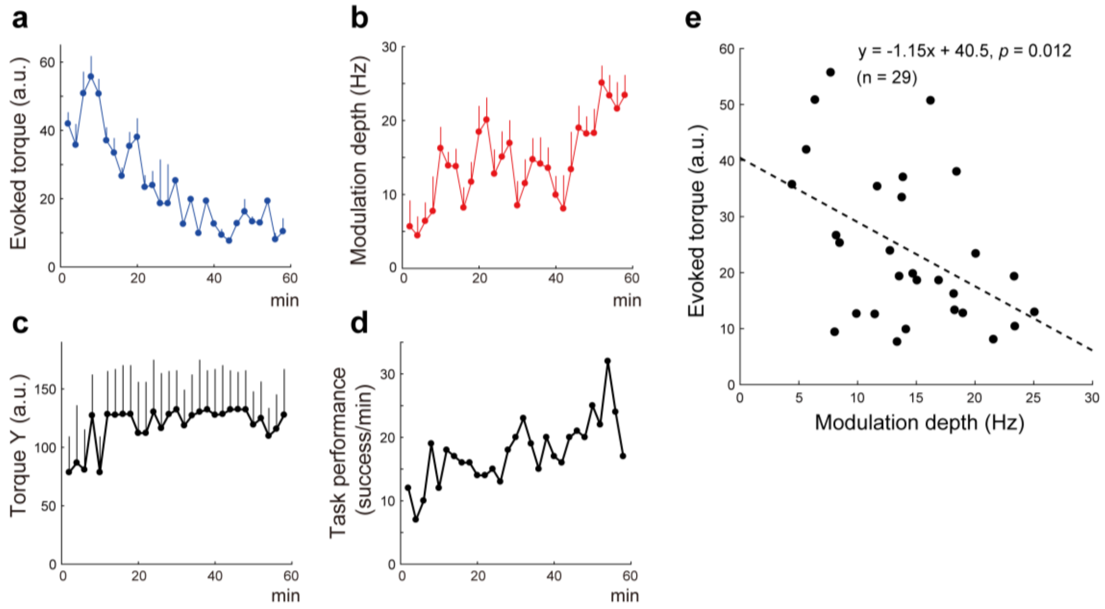

Supplementary Figure 10. **Gamma activity compensates for muscle fatigue. a**: Plots of evoked torque induced by electrical stimulation during 2 min bins in a long-lasting-session. The input electrode was in the digit-innervated area within M1. Electrical stimuli were applied to the extensor carpi radialis (ECR) muscle. Data indicate means plus standard errors. **b**: Plot of average high-gamma modulation depths (MDs) for each 2 min time bin. **c**: Torque measurements for each 2 min time bin. **d**: Plot of task performance for each 2 min bin. **e**: A significant correlation between evoked torque and high-gamma MD is shown by the dotted line (Pearson correlation coefficient, n = 29, R = -0.461, P = 0.012). Data were obtained from Monkey M (ECoG array). Data in panels **a-d** represent means and standard errors.

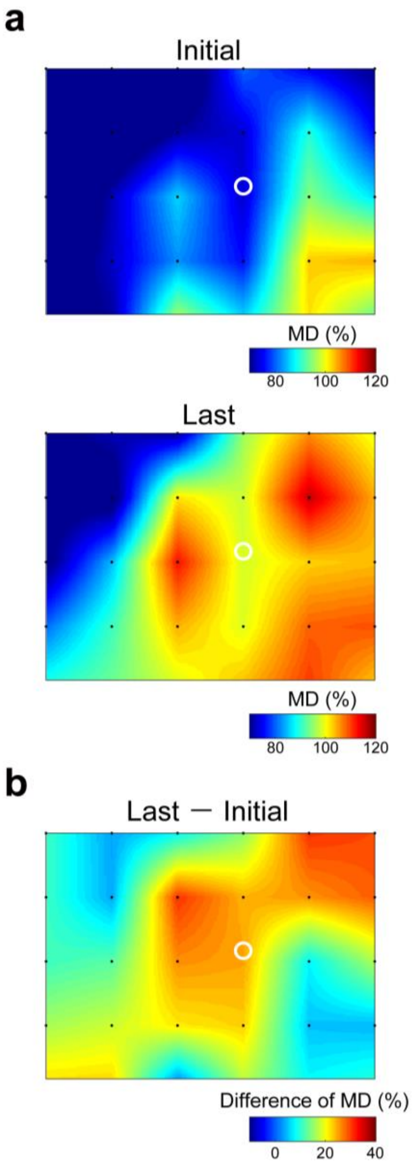

Supplementary Figure 11. **Targeted cortical adaptation during learning with an ACMC in Monkey TE. a:** Topographic map of the MDs of high-gamma power during the initial (a) and last phases (b). The white circle indicates the input electrode used for the ACMC. Electrodes not used for the ACMC are shown as black dots. Electrical stimulation was applied transcutaneously to the wrist extensor muscles. **b:** Changes in the spatial distribution of MD determined by subtracting the MD during the initial phase from the MD during the last phase.

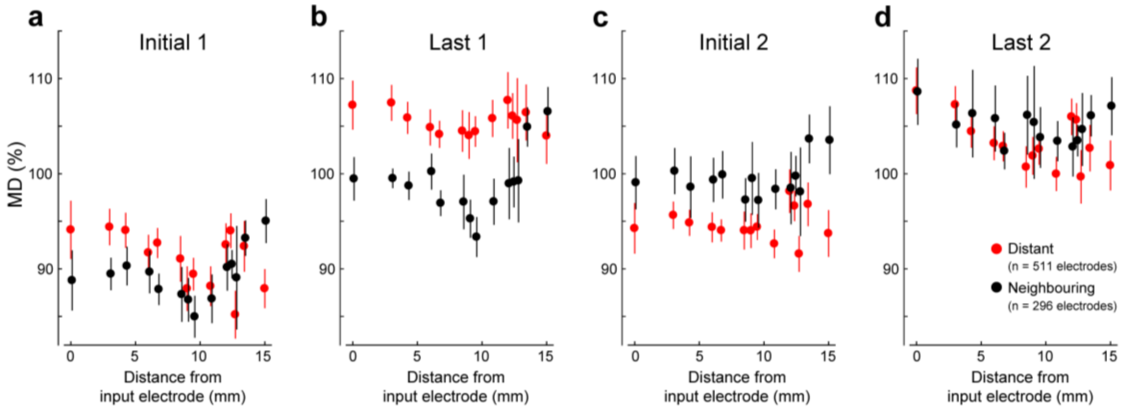

Supplementary Figure 12. The relationship between MD and the distance from the input electrode. **a** and **b** are Initial and Last phase in Session 1 respectively. **c** and **d** are Initial and Last phase in Session 2 respectively (N = 2 monkeys [Monkey TE and M], Distant [red], n = 511 electrodes; Neighbouring [black], n = 296 electrodes). **a**: Initial 1, Two-way ANOVA; [Distance] $F_{(13, 779)} = 1.58$, $P = 8.57 \times 10^{-2}$, not significant. [Switching] $F_{(1, 779)} = 3.69$, $P = 5.52 \times 10^{-2}$, not significant. **b**: Last 1, Two-way ANOVA; [Distance] $F_{(13, 779)} = 1.39$, $P = 0.16$, not significant. [Switching] $F_{(1, 779)} = 0.46$, $P = 2.01 \times 10^{-11}$. **c**: Initial 2, Two-way ANOVA; [Distance] $F_{(13, 779)} = 0.70$, $P = 0.77$, not significant. [Switching] $F_{(1, 779)} = 27.91$, $P = 1.65 \times 10^{-7}$. **d**: Last 2, Two-way ANOVA; [Distance] $F_{(13, 779)} = 0.74$, $P = 0.73$, not significant. [Switching] $F_{(1, 779)} = 2.74$, $P = 9.82 \times 10^{-2}$, not significant. Detailed statistical results are shown in the source data. Data indicate means and standard errors.

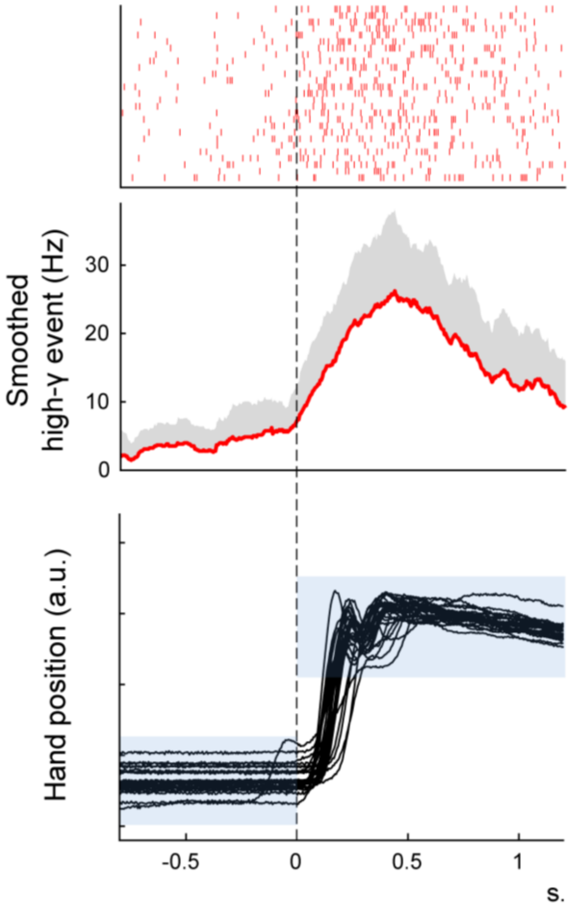

Supplementary Fig. 13. Modulation of high-gamma activity during position-tracking task in an intact animal. Top, raster plot of high-gamma episodes. Middle, plot of the average rate of occurrence of high-gamma episodes. Bottom, plot of hand position. The blue-shaded rectangles represent hand position targets. All plots are aligned to the time of target appearance, indicated by the vertical dotted line. Data obtained from Monkey TA before stroke induction. n = 27 trials. Cortical signals were recorded by microelectorodes.
