# Supplementary material for: Bypassing stroke-damaged neural pathways via a neural interface induces targeted cortical adaptation
Source: Nat Commun. 2019 Oct 16;10:4699. doi: 10.1038/s41467-019-12647-y (PMC6796004; doi:10.1038/s41467-019-12647-y)
Supplement: Supplementary file 3 — Reporting Summary [file 41467_2019_12647_MOESM3_ESM.pdf]

nature research

Corresponding author(s): Yukio Nishimura

Last updated by author(s): Aug 22, 2019

Reporting Summary

Nature Research wishes to improve the reproducibility of the work that we publish. This form provides structure for consistency and transparency in reporting. For further information on Nature Research policies, see Authors & Referees and the Editorial Policy Checklist .

Statistics

For all statistical analyses, confirm that the following items are present in the figure legend, table legend, main text, or Methods section.

n/a	Confirmed	
☐	☒	The exact sample size (*n*) for each experimental group/condition, given as a discrete number and unit of measurement
☐	☒	A statement on whether measurements were taken from distinct samples or whether the same sample was measured repeatedly
☐	☒	The statistical test(s) used AND whether they are one- or two-sided<br>*Only common tests should be described solely by name; describe more complex techniques in the Methods section.*
☐	☒	A description of all covariates tested
☐	☒	A description of any assumptions or corrections, such as tests of normality and adjustment for multiple comparisons
☐	☒	A full description of the statistical parameters including central tendency (e.g. means) or other basic estimates (e.g. regression coefficient) AND variation (e.g. standard deviation) or associated estimates of uncertainty (e.g. confidence intervals)
☐	☒	For null hypothesis testing, the test statistic (e.g. *F*, *t*, *r*) with confidence intervals, effect sizes, degrees of freedom and *P* value noted<br>*Give P values as exact values whenever suitable.*
☒	☐	For Bayesian analysis, information on the choice of priors and Markov chain Monte Carlo settings
☒	☐	For hierarchical and complex designs, identification of the appropriate level for tests and full reporting of outcomes
☐	☒	Estimates of effect sizes (e.g. Cohen's *d*, Pearson's *r*), indicating how they were calculated

Our web collection on statistics for biologists contains articles on many of the points above.

Software and code

Policy information about availability of computer code

Data collection	ECoG signals in monkey TE and M were recorded with a Cerebus™ data acquisition system (Blackrock Microsystems, Salt Lake City, UT, USA). Local field potential signals in monkey TA were recorded with a CED 1401 data acquisition system (CED, Cambridge, UK).
Data analysis	We performed Data analysis using custom software written in MATLAB (The MathWorks Inc., MA, USA). Statistical analysis were performed using MATLAB statistical tool box.

For manuscripts utilizing custom algorithms or software that are central to the research but not yet described in published literature, software must be made available to editors/reviewers. We strongly encourage code deposition in a community repository (e.g. GitHub). See the Nature Research guidelines for submitting code & software for further information.

Data

Policy information about availability of data

All manuscripts must include a data availability statement. This statement should provide the following information, where applicable:

- Accession codes, unique identifiers, or web links for publicly available datasets
- A list of figures that have associated raw data
- A description of any restrictions on data availability

Data supporting the findings of this study are available from the corresponding authors upon reasonable request.

Field-specific reporting

Please select the one below that is the best fit for your research. If you are not sure, read the appropriate sections before making your selection.

☒ Life sciences ☐ Behavioural & social sciences ☐ Ecological, evolutionary & environmental sciences

Life sciences study design

All studies must disclose on these points even when the disclosure is negative.

Sample size	No statistical methods were used for predetermining sample size. We used a sample size that is comparable to previously published studies in the field.
Data exclusions	Neither data nor animals were excluded.
Replication	All of three monkeys with subcortical stroke performed behavioral task. Experiments were repeated using 92 different pairs of cortical sites and muscles. Main findings were highly reproducible as you see in results.
Randomization	The experiments were not randomized. Because of all of three monkeys displayed hemiparalysis due to subcortical stroke, the experimenter could detect that the monkeys are stroke monkeys.
Blinding	Blinding was not used. Because of all of three monkeys displayed hemiparalysis due to subcortical stroke, the experimenter could detect that the monkeys are stroke monkeys.

Reporting for specific materials, systems and methods

We require information from authors about some types of materials, experimental systems and methods used in many studies. Here, indicate whether each material, system or method listed is relevant to your study. If you are not sure if a list item applies to your research, read the appropriate section before selecting a response.

Materials & experimental systems

n/a	Involved in the study
☒	☐ Antibodies
☒	☐ Eukaryotic cell lines
☒	☐ Palaeontology
☐	☒ Animals and other organisms
☒	☐ Human research participants
☒	☐ Clinical data

Methods

n/a	Involved in the study
☒	☐ ChIP-seq
☒	☐ Flow cytometry
☐	☒ MRI-based neuroimaging

Animals and other organisms

Policy information about studies involving animals; ARRIVE guidelines recommended for reporting animal research

Laboratory animals	The experiments were performed using 3 male macaque monkeys.<br>2 Macaca fuscata: Monkey M, 9.0 kg and Monkey TA, 7.5 kg; 1 Macaca mulatta; Monkey TE, 9.8 kg
Wild animals	N.A.
Field-collected samples	N.A.
Ethics oversight	All experimental procedures were performed in accordance with the guidelines of the National Institutes of Health and the Ministry of Education, Culture, Sports, Science, and Technology (MEXT) of Japan, and were approved by the Institutional Animal Care and Use Committee of the National Institutes of Natural Sciences (Approval No. 14A125).

Note that full information on the approval of the study protocol must also be provided in the manuscript.

Magnetic resonance imaging

Experimental design

Design type	Structural MRI under deep anaesthesia
Design specifications	Scan time: 5 min scan, number of block: 4, number of average: 4
Behavioral performance measures	N.A.

Acquisition

Imaging type(s): Structural

Field strength: 3 tesla

Sequence & imaging parameters: T2-weighted MRI was performed using a two-dimensional turbo spine echo sequence (0.5 × 0.5 mm, 40 slices, slice thickness = 1.0 mm, TR/TE = 7880/19 ms).

Area of acquisition: A whole brain scan

Diffusion MRI: ☐ Used ☒ Not used

Preprocessing

Preprocessing software: N.A.

Normalization: N.A.

Normalization template: N.A.

Noise and artifact removal: N.A.

Volume censoring: N.A.

Statistical modeling & inference

Model type and settings: N.A.

Effect(s) tested: N.A.

Specify type of analysis: ☒ Whole brain ☐ ROI-based ☐ Both

Statistic type for inference (See Eklund et al. 2016): N.A.

Correction: N.A.

Models & analysis

n/a	Involved in the study
☒	☐ Functional and/or effective connectivity
☒	☐ Graph analysis
☒	☐ Multivariate modeling or predictive analysis
