# Supplementary material for: Bypassing stroke-damaged neural pathways via a neural interface induces targeted cortical adaptation
Source: Nat Commun. 2019 Oct 16;10:4699. doi: 10.1038/s41467-019-12647-y (PMC6796004; doi:10.1038/s41467-019-12647-y)
Supplement: Supplementary file 2 — Peer Review File [file 41467_2019_12647_MOESM2_ESM.pdf]

Editorial Note: This manuscript has been previously reviewed at another journal that is not operating a transparent peer review scheme. This document only contains reviewer comments and rebuttal letters for versions considered at *Nature Communications.*

REVIEWERS' COMMENTS:

Reviewer #1 (Remarks to the Author):

All of reviewer #1 comments are adequately addressed.

Reviewer #3 comment 1: I agree with the authors that the work is correctly represented, and the recent revision makes this abundantly clear. I am glad to see that the the recent (since 2016) citations have been added, most importantly Ajiboye et al, 2017, as the Coutine 2018 paper in humans is much less relevant here (epidural stimulation without any BCI). There is a Courtine paper using BCI to control lumbar spinal stimulation (Capogrosso et al. 2016), that may or may not be cited – I'll leave this up to the Authors to decide.

Comment 2: Again, there reviewer is mistaken in that Courtine 2018 did not involve any BCI. They also do not credit the novelty of using alternative brain areas, something that is critical after stroke damages M1 used in most all of the other studies cited. This reviewer also exaggerates the finding of Ethier/Miller, the grasp was just simple 1D control. As the authors correctly respond, there are significant advantages to demonstrating ECoG control of a BCI, especially in the case of stroke where penetrating electrodes may be contraindicated due to risk of bleeding.

Comment 3: I agree with the authors that the level of difficulty and the response time of muscle stimulation in this manuscript creates a much more complex and challenging paradigm, so the results should not be compared to the Degenhart work. The animals here also learn a new control site frequently and rapidly, which is a significant advance on the state of the art.

Comment 4: I agree with the authors' response here as well.

Comment 5: Rigor and time to target. This is standard practice in many BCI studies, and I agree with the authors response.

Comment 6: I do not completely understand how the reviewer can decouple BCI decoder performance from the animal's performance, especially when the animal is rapidly learning new tasks. The authors have responded well to this confusing criticism.

Comment 7: SEM is standard in the field when combining across sites/sessions or animals.

Comment 8: FES movements look jerky no matter how they are controlled. The authors responded/revised well here.

Comment 9: The authors have responded well. Only a small subset of the filed cares about modulation depth anymore, but they have added it.

Comment 10: SEM again, I agree with the authors response.

Comment 11: While there is slight variability in the training/implant, three monkeys are presented with exceeds the threshold for primate studies, and the data are robust/consistent across animals.

Comment 12: Ethics concern. I do not share the concerns about ethics raised by the reviewer. The results are novel and informative, and all procedures were pre-approved by the animal ethics committee, regardless of the outcome of the study.

Sincerely,
Chet Moritz

REVIEWERS' COMMENTS:

Reviewer #1 (Remarks to the Author):

All of reviewer #1 comments are adequately addressed.
Response: Thanks.

Reviewer #3 comment 1: I agree with the authors that the work is correctly represented, and the recent revision makes this abundantly clear. I am glad to see that the the recent (since 2016) citations have been added, most importantly Ajiboye et al, 2017, as the Coutine 2018 paper in humans is much less relevant here (epidural stimulation without any BCI). There is a Courtine paper using BCI to control lumbar spinal stimulation (Capogrosso et al. 2016), that may or may not be cited – I'll leave this up to the Authors to decide.
Response: Capogrosso et al. 2016 was not cited, since it does not involve any BCI.

Comment 2: Again, there reviewer is mistaken in that Courtine 2018 did not involve any BCI. They also do not credit the novelty of using alternative brain areas, something that is critical after stroke damages M1 used in most all of the other studies cited. This reviewer also exaggerates the finding of Ethier/Miller, the grasp was just simple 1D control. As the authors correctly respond, there are significant advantages to demonstrating ECoG control of a BCI, especially in the case of stroke where penetrating electrodes may be contraindicated due to risk of bleeding.
Response: Thanks.

Comment 3: I agree with the authors that the level of difficulty and the response time of muscle stimulation in this manuscript creates a much more complex and challenging paradigm, so the results should not be compared to the Degenhart work. The animals here also learn a new control site frequently and rapidly, which is a significant advance on the state of the art.
Response: Thanks.

Comment 4: I agree with the authors' response here as well.

Response: Thanks.

Comment 5: Rigor and time to target. This is standard practice in many BCI studies, and I agree with the authors response.

Response: Thanks.

Comment 6: I do not completely understand how the reviewer can decouple BCI decoder performance from the animal's performance, especially when the animal is rapidly learning new tasks. The authors have responded well to this confusing criticism.

Response: Thanks.

Comment 7: SEM is standard in the field when combining across sites/sessions or animals.

Response: Thanks.

Comment 8: FES movements look jerky no matter how they are controlled. The authors responded/revised well here.

Response: Thanks.

Comment 9: The authors have responded well. Only a small subset of the filed cares about modulation depth anymore, but they have added it.

Response: Thanks.

Comment 10: SEM again, I agree with the authors response.

Response: Thanks.

Comment 11: While there is slight variability in the training/implant, three monkeys are presented with exceeds the threshold for primate studies, and the data are robust/consistent across animals.

Response: Thanks.

Comment 12: Ethics concern. I do not share the concerns about ethics raised by the reviewer. The results are novel and informative, and all procedures were pre-approved by the animal ethics committee, regardless of the outcome of the study.

Response: Thanks.

Sincerely,
Chet Moritz

Best regards,
Yukio Nishimura
